# Capacity factors for electrical power generation from renewable and nonrenewable sources

**DOI:** 10.1073/pnas.2205429119

**Published:** 2022-12-20

**Authors:** Natanael Bolson, Pedro Prieto, Tadeusz Patzek

**Affiliations:** ^a^Ali I. Al-Naimi Petroleum Engineering Research Center, King Abdullah University of Science and Technology, Thuwal 23955-6900, Saudi Arabia

**Keywords:** electricity sources, wind, solar, energy transition, renewable energy

## Abstract

Given the dire consequences of climate change and the war in Ukraine, decarbonization of electrical power systems around the world must be accomplished, while avoiding recurring blackouts. A good understanding of performance and reliability of different power sources underpins this endeavor. As an energy transition involves different societal sectors, we must adopt a simple and efficient way of communicating the transition’s key indicators. Capacity factor (CF) is a direct measure of the efficacy of a power generation system and of the costs of power produced. Since the year 2000, the explosive expansion of solar PV and wind power made their CFs more reliable. Knowing the long-time average CFs of different electricity sources allows one to calculate directly the nominal capacity required to replace the current fossil fuel mix for electricity generation or expansion to meet future demand. CFs are straightforwardly calculated, but they are rooted in real performance, not in modeling or wishful thinking. Based on the current average CFs, replacing 1 W of fossil electricity generation capacity requires installation of 4 W solar PV or 2 W of wind power. An expansion of the current energy mix requires installing 8.8 W of solar PV or 4.3 W of wind power.

## Significance

Capacity factor (CF) of an electrical generation plant is a direct measurement of the efficacy of this plant, or all power plants in a country, region, or the world. CF measures directly how much electrical power is produced by a plant relative to how much could possibly be produced at peak capacity. In view of a dire need to decarbonize and transition to clean energy, long-time average CFs provide a key component of reliable, unbiased insights into what is required to replace the current fossil fuel mix (coal, natural gas, and oil). CFs also are needed for an accurate quantification of the nominal generation capacity needed to replace and expand the current electricity infrastructure.

Greenhouse gas emissions are the driving force behind climate change (1), which threatens biodiversity (2), food security (3), and cultural diversity (4). The main source of carbon emissions in electricity generation is the current mixture of inputs (5). The current state of affairs demands an energy transition, but numerous challenges emerge (6–8). Such a transition implicates different societies in different ways (9–11), but even the conservative United States wants a decarbonized future (12). Actions toward a sustainable future have been taken at different scales from city (13) to country level (14).

The current energy system in place has a rigid structure with a modus operandi of “winner takes all” that hampers the establishment of alternatives (15). Policies can be implemented to overcome the status quo. However, unintended consequences can arise, e.g., carbon pricing policies tend to incentivize optimization of the current energy system instead of the required transformations to achieve a decarbonized one (16). This conclusion is not universally accepted (17–19). It seems, however, that renewables will lead the energy transition and solar photovoltaics will play a key role (20–22).

Engaging different players in a society for the energy transition is essential. However, the diversity of stakeholders creates communication barriers, particularly when technical details are transmitted to a broad audience. The war in Ukraine and insufficient natural gas supply in Europe have added painful urgency to clear and truthful communication of the potential pitfalls of any energy transition that boil down to the clear understanding of what the different components of electricity generation systems can and cannot do. Regarding power-generation efficiencies of different sources, the use of CF is an excellent tool to connect with a broad set of audiences.

CF is a measure of a power plant efficacy (23). In short, it is an indicator of how fully the power plant is used, relative to its thermodynamic and technological constraints and required spare capacity (24). For all technologies, CFs have typical values for a set time interval and input (a fuel, light, water, or wind).

An electrical power plant’s CF gives this plant’s average output relative to its maximum capacity. This could be quite misleading for renewables. If a plant works at 50% of nominal capacity, its CF is 0.5. This does not mean that the plant worked 12 h at full capacity and was off over the remaining 12 h. This plant could be down for different reasons such as repairs, maintenance, refueling, or intermittency for renewables. Despite its limitations, CF is a straightforward indicator that can be easily calculated and predict the amount of electricity that will be obtained on average from a specific nominal capacity installed.

Comparing CFs across different technologies can be tricky, mainly when some are well established and mature, while others are at pilot-scale or not fully deployed. In the last two decades, solar PV and wind have been growing exponentially. This explosive growth allows one to obtain reliably their CFs. When a technology is more established, the effects of pilot plants, learning curves (25–28), or optimal sites no longer dominate, giving reliable estimates about that technology’s performance. Field tests are the ultimate answer. Theoretical estimates can differ significantly from the measured ones. Such discrepancies have been presented for wind (29) and concentrated solar power plants (30) (*SI Appendix*, section 10). Knowing the real value of CFs is fundamental to estimating costs, power production, and the future roles of specific technologies.

In this work, we analyze the average CFs of different electricity sources (i.e., biomass, fossil fuels, geothermal heat, water, uranium, solar light, and wind) over the period 2000–2017. Global and regional values are estimated to highlight the differences in the performance of different technologies. These average CF values are then used to calculate the required nominal capacity to be installed in the future for our unavoidable energy transition.

## Capacity Factor (CF)

CF is the ratio of the actual average electrical power a plant delivers over time to the nominal power it is capable of delivering at peak conditions. Through weighting the CF by the share of electricity generated, we reduce the influence of countries that do not have a significant capacity for a specific technology. It could be because they are at the initial stages of adopting the technology, or it is not feasible, i.e., no plans exist for expansion after pilot plants. Also, initially, the best places for production are targeted, but the early projects or pilot plants can skew CF estimates. As the piloted technology matures and spreads, a more reliable value of CF is obtained.

**Fig. 1.**
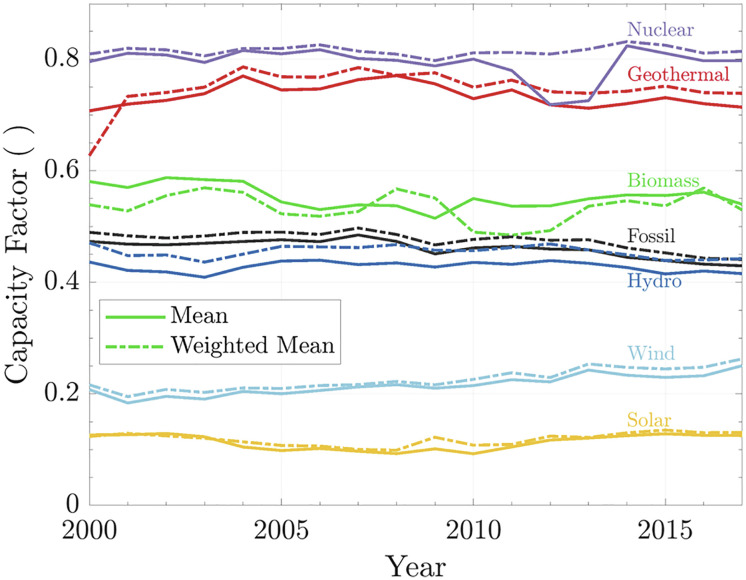
World capacity factor. The solid line is for the capacity factor, and the dashed line shows weighted capacity factor for the respective year.

Fig. 1 shows the CFs of different technologies. When the dashed line is above the solid line, it means that the big producers have a higher CF than the average, see, e.g., hydropower.

It is important to mention that CF fluctuations can occur for many reasons. For renewables that rely on weather conditions, an atypical year can interfere (e.g., a severe drought in Brazil in 2021). In the case of sources that depend only on demand (e.g., fossil fuels), excess production from renewables could induce reductions of the fossil power CFs. Another example is nuclear power after the Fukushima incident in 2011, which triggered a wave of shutdowns and maintenance of nuclear reactors worldwide (31).

**Fig. 2.**
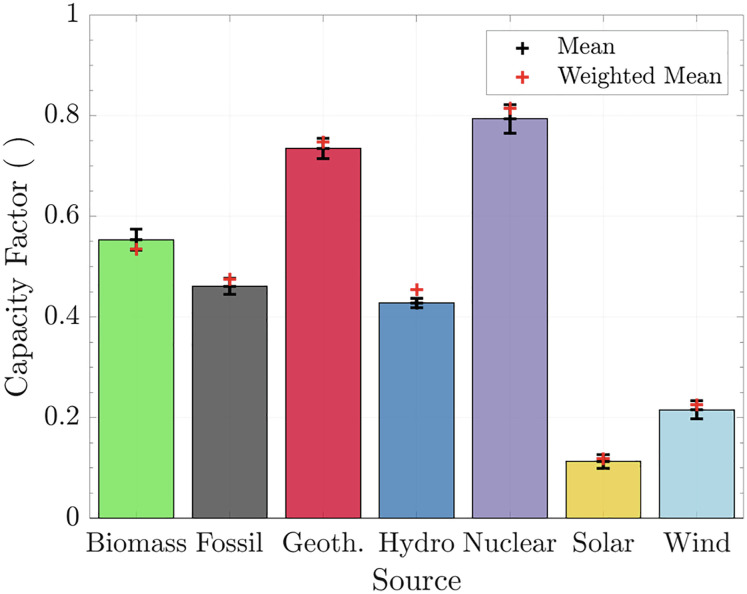
World’s mean and weighted mean capacity factors. The mean values correspond to the 2000–2017 period. The error bar is the SD.

Fig. 2 shows the means and weighted means of CFs for the world. These values are suitable for evaluating the overall performance of a specific technology. With the maturation and spread of solar PV and wind power plants, we may already know their reliable mean CF values of 0.11 and 0.22, respectively. The mean and weighted mean have similar values for all sources, which implies that globally the impact of big producers is insignificant. *SI Appendix*, Fig. S2 shows the boxplot for CFs of the world regions, and *SI Appendix*, Fig. S3 gives a detailed perspective of the composition of fossil fuels used to generate electricity.

Fig. 2, in other words, could be interpreted as the efficacy of the nominal installed power, e.g., installing 5 W of wind power, is equivalent to 1 W of an ideal generator. It is important to emphasize that capacity factor (CF) does not capture variance and intermittence of energy generation, which in the case of some renewables means that operation can oscillate between maximum and zero power in a few hours.

Table 1 shows the CFs for all the technologies and regions analyzed. While globally we do not observe significant differences between the mean and weighted mean; for some regions, these differences are significant. Some regions have big energy players who drive the weighted mean, as in Oceania dominated by Australia. Another example is hydropower in Latin America, in which Brazil is responsible for half of all electricity generated from water.

**Table 1.** Summary of capacity factor values

	Biomass		Fossil		Geothermal		Hydro		Nuclear		Solar		Wind	
Region	Mean	W. M.	Mean	W. M.	Mean	W. M.	Mean	W. M.	Mean	W. M.	Mean	W. M.	Mean	W. M.
Africa	0.32	0.43	0.31	0.57	0.56	0.74	0.44	0.55	0.80	0.80	0.17	0.17	0.26	0.27
Asia	0.37	0.60	0.41	0.51	0.65	0.70	0.37	0.39	0.72	0.76	0.14	0.12	0.23	0.19
CIS	0.34	0.31	0.32	0.43	0.60	0.60	0.37	0.40	0.72	0.76	0.10	0.07	0.12	0.12
Europe	0.53	0.59	0.37	0.44	0.63	0.86	0.38	0.44	0.83	0.79	0.10	0.10	0.21	0.21
L. Am.	0.37	0.45	0.36	0.45	0.67	0.76	0.47	0.53	0.77	0.79	0.20	0.12	0.27	0.31
MENA	0.47	0.52	0.49	0.49	–	–	0.27	0.36	0.44	0.44	0.11	0.11	0.27	0.33
N. Am.	0.62	0.64	0.44	0.43	0.72	0.72	0.48	0.49	0.85	0.89	0.14	0.15	0.26	0.27
Oceania	0.32	0.45	0.40	0.52	0.87	0.87	0.45	0.43	–	–	0.15	0.14	0.22	0.31
World	0.55	0.53	0.46	0.48	0.74	0.75	0.43	0.45	0.79	0.81	0.11	0.12	0.22	0.23

W. M. is the acronym for the Weighted Mean.

Also, Table 1 allows us to compare how specific technologies perform in different regions. For example, solar PV is more productive in MENA than in Europe. Understanding these differences is crucial to proposing any global energy transition strategy as different regions have their strengths and weakness. A comparison of all the regions is available in *SI Appendix*, Fig. S7. Also, being aware of each region’s values can provide a better perspective than the global values, avoiding overestimation or underestimation. In addition, the values for individual countries are shown in supplementary *SI Appendix*, Table S1. Supplementary information for the share of electricity generated, nominal capacity installed, historical values, and box-plot of CF are available in *SI Appendix*, Figs. S8–S28.

## Energy Transition and Technology Replacement

**Fig. 3.**
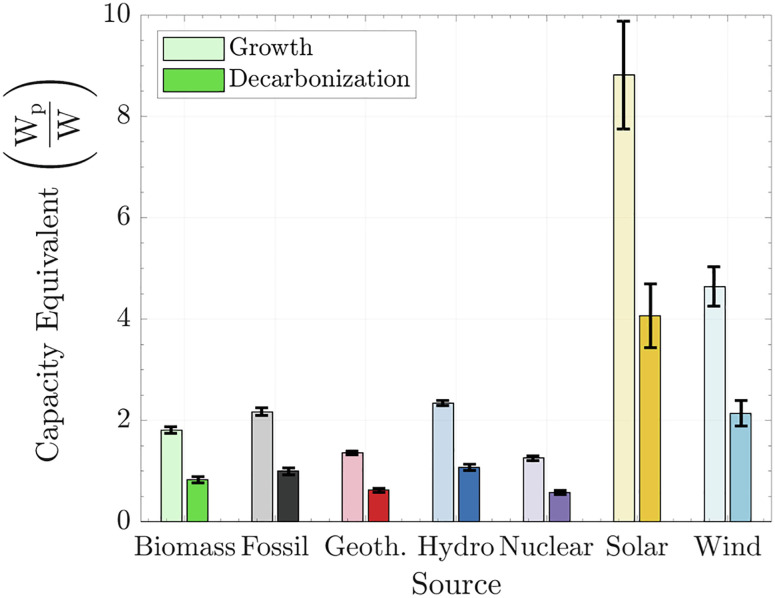
Capacity requirements for an energy transition. W_p_ represents the nominal capacity that must be installed to power a unit of power output continuously (W)—limitations such as intermittence of losses due to storage are neglected. The Growth scenario represents an expansion of the current power sector in place to supply an increase in power consumption. The Decarbonization scenario is the replacement of fossil fuels in the electrical power sector by alternative sources; in this case, total power supply remains constant. Error bars represent one SD.

Knowing the mean CF of a particular technology provides critical insights needed to design and expand the infrastructure required to supply electricity. Fig. 3 illustrates the nominal capacity (W_p_) equivalent that must be deployed to supply a unit of power demand (W). The Growth scenario represents an expansion of the current power sector in place to satisfy an increase in power consumption (i.e., power addition to the system). The Decarbonization scenario is the replacement of fossil fuels in the power sector by alternative sources; in this case, the total power supply remains constant (this is a true power transition). An energy transition is only achieved when a replacement occurs (Decarbonization). Otherwise, we would just be adding to the system-in-place (Growth) (7).

Fig. 3 shows that the Growth scenario requires practically twice the nominal capacity required for Decarbonization. The reason is that in the Decarbonization scenario, we are replacing a technology that has the global CF of 0.46, i.e., we assume that the global fossil fuel power generation operates continuously at roughly half capacity (and presumably satisfies power peaks).

Based on the current CFs, an energy transition toward renewables will require a massive infrastructure and nominal capacity to be built and installed. To replace the current fossil capacity that generates 4 TW_*e*_ with solar PV and wind at a 1:1 ratio, we must install 12 TW_*e*_ from these renewables. This estimate applies to a replacement-only scenario with no growth, also assuming that the 0.5 CF for fossil plants is all waste and not needed to provide peak load. For an expansion scenario, each 1 TW_*e*_ of fossil fuel electricity displaced by 1:1 solar PV and wind would require 6.5 TW_*e*_ from these renewables.

**Fig. 4.**
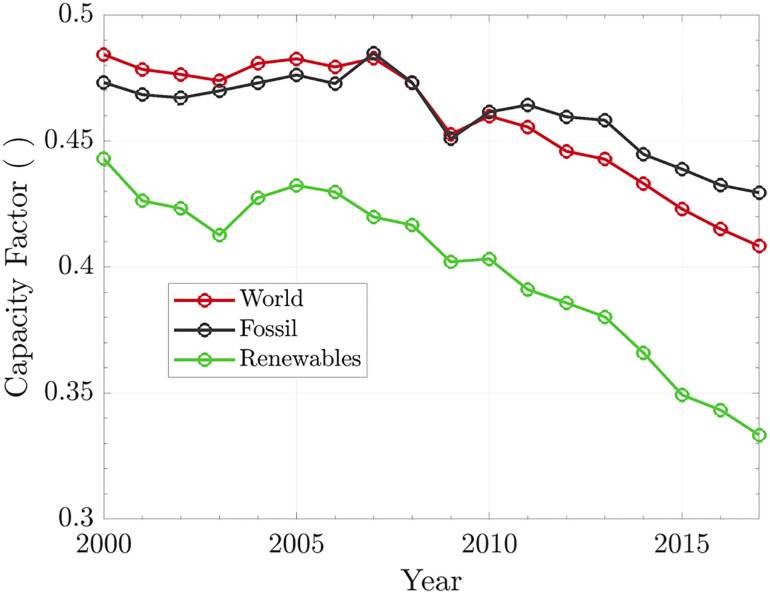
The historical values of capacity factors. “World” includes all the nominal capacity installed to generate electricity; “Fossil” includes coal, natural gas, and oil; “Renewables” encompass all renewables sources.

Fig. 4 shows that a gradual growth of the share of renewables in the world’s electrical power mix has caused the CFs of the fossil fuel plants and renewable plants to decline with time. This means that we are increasing the overall capacity of electricity generation, but at the same time, the emerging new system has more idle capacity. We should be aware of this problem because it is intrinsic to the modern technology mix.

The declining CF for fossil fuels is intriguing. The first hypothesis is that it could be caused by the replacement of fossil fuels with renewables. However, the installed fossil fuel capacity is still growing, which contradicts this scenario (*SI Appendix*, Fig. S4). The second hypothesis intimates a mismatch between the foreseen and actual electricity demand. A fossil power plant takes on average five years to build (19). Forecasting electricity demand involves several factors, but usually, it is well-correlated with the gross domestic product (GDP). It seems that a mismatch between expected and real GDP growth could explain this decline. The World Economic Outlook from the International Monetary Fund (32) estimated that the world annual GDP growth would be near to 4.5%. This growth rate happened only in 2010 (5.4%) and 2011 (4.3%), but for the following years until 2017, the average was 3.5% (33). Knowing that, on average, it takes 5 y to build a fossil power plant (19), the capacity added was envisioning a higher electricity demand (*SI Appendix*, Fig. S5). Although undesired, this condition is not surprising. As a precautionary measure in power supply, it is a safe strategy.

**Fig. 5.**
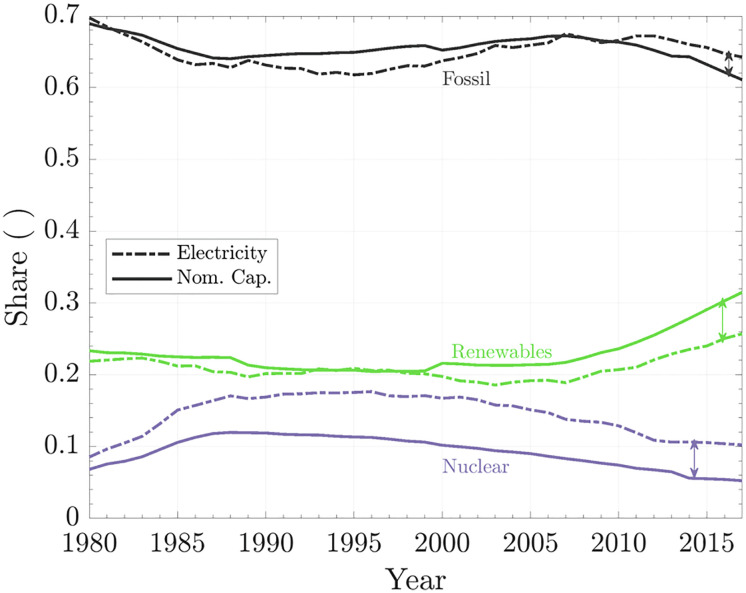
Nominal capacity and electricity generation. The solid lines show the shares of nominal capacity installed, and the dashed lines show the shares of electricity generated.

Fig. 5 shows the share of nominal capacity (solid line) and net electricity generation (dashed line) for the global fossil, nuclear, and renewable power plants. The electrical power mix has an average CF that depends on the shares of each source. The current world CF is 0.41, which is based on a consideration of the total installed capacity independent of the source and the total electricity generation. When comparing the share of electricity generated and nominal capacity, three conditions are possible:

1. Share of nominal capacity and electricity generation overlap. In this case, the share of capacity installed is the same as the system’s CF (see fossil Fig. 5).
2. Share of nominal capacity is greater than the electricity generation. This case implies that this technology’s CF is lower than the system’s average CF (see renewables, Fig. 5).
3. Share of nominal capacity is smaller than the electricity generation. This case means that the technology has a higher CF than the system (see nuclear, Fig. 5).

When we consider the three possible conditions and the results shown in Fig. 5, we notice a bifurcation in the share of renewables. On the one hand, their installed capacity has increased more rapidly than those of the other sources, but on the other hand, the share of the global electricity generated by renewables has lagged increasingly (see the arrows in Fig. 5). Thus, the addition of renewables to the energy mix is lowering the system’s CF and explains why, after the year 2005, the fossil fuel power plants switched from the condition described in ref. 2 to that in ref. 3.

**Fig. 6.**
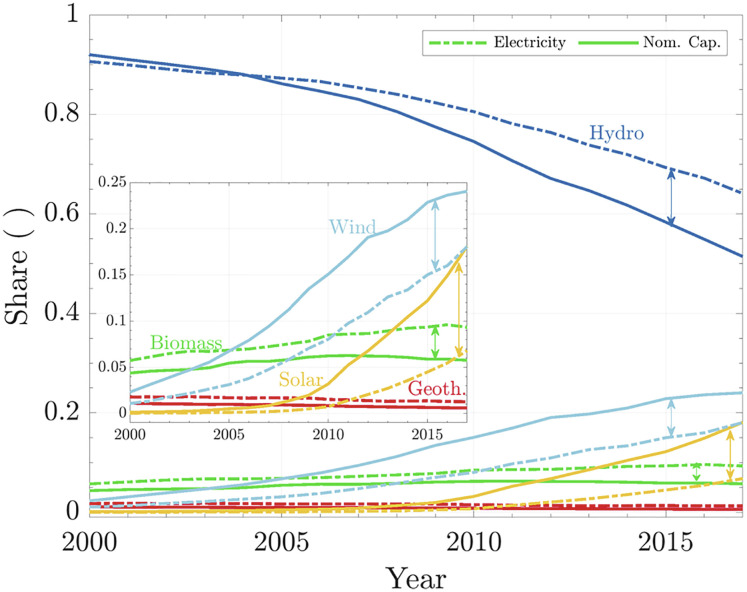
Detailed nominal capacity installed and electricity generated by renewables. The solid lines show the shares of nominal capacity installed, and the dashed lines show the share of electricity generated.

As previously mentioned, after the year 2000, our energy mix started to change due to an aggressive expansion of renewables, mainly solar and wind power. Fig. 6 shows the nominal capacity and electricity generated by renewable sources. Before the year 2000, the energy mix was practically composed of biomass, geothermal, and hydropower. Nowadays, solar and wind have a significant presence. Consequently, the overall CF for renewables dropped from 0.44 to 0.33 over the last two decades (Fig. 4).

The critical observation from Fig. 6 is that the share of installed capacity does not correspond to the share of electricity generated. One should be aware of this fact when applauding the increasing presence of renewables in the energy mix. As more solar and wind are added to the energy system, they will reduce the global CF. This fact should not discourage the mandatory energy transition, but it foreshadows the magnitude of the challenge that lies ahead.

An energy transition scenario that requires 12 TW of solar and wind to replace the fossil fuel energy mix presents several challenges. It will triple peak demands on the energy infrastructure from the power transmission lines and storage, to generation. This transition scenario involves a giant new infrastructure at all levels that usually is not accounted for ref. (34). Copper demand could become a problem. Solar photovoltaic systems use 5 tons of copper per MW installed (35), while wind turbines require up to 5 tons of copper per MW installed onshore (36) and up to 10 tons for offshore systems (37). Thus, our energy transition scenario could easily require extra 75 million tonnes of copper. This estimate ignores most of the new infrastructure required to accommodate the growing world population and electrifying industry. For reference, the current global extraction rate is 20 million tonnes of copper per year, with current reserves estimated at 870 million tonnes (38).

Furthermore, the ubiquity of intermittent renewables in the power system creates new challenges for grid management. First, the peaks of production or dispatch limitations could result in curtailment of electricity, reducing the system efficiency (39). Second, energy storage is currently a great challenge; while there is no dominant technology, many options are available, improving, or are being developed (40–42).

## Solar Power

Solar PV is expected to play a major role in any energy transition scenario (43). Thus, to gain deeper insights, we analyzed four uniquely different PV installations, see *SI Appendix*, sections 9 and 10 for details.

When we consider the performance of a solar photovoltaic device, we must think beyond the incident solar irradiance. First, the temperature of the photovoltaic cells affects the power output. As this temperature increases with global warming (44), the power output decreases proportionally (45). Conversely, adoption of a sun-tracking system can increase the power output from 1545, when compared with the fixed panel systems (46, 47). However, the performance of the sun-tracking systems depends on ambient conditions. They are most effective in cold and cloudy countries, rather than in a hot climate (48). Sun-tracking in a hot environment increases the incident radiation over the panel, increasing the power output. However, it also increases the panel’s temperature, reducing the power output. The sun-tracking PV arrays have higher operational and maintenance costs (49). In some cases, the net power output gain does not compensate for the energy cost of the tracking system. Also, in windy places, costs increase further due to reinforced structures needed to withstand the wind load and energy output reduction due to the array’s flag position to avoid mechanical damage. Another issue associated with photovoltaics is dust, 4month of dust can reduce the power output of a solar panel by 40 (50). Soiling losses over a dry summer can reduce the power output of a solar panel by 20 (51). Current models neglect the impact of dust on the power output of solar panels, which can lead to quite misleading CF estimates.

**Fig. 7.**
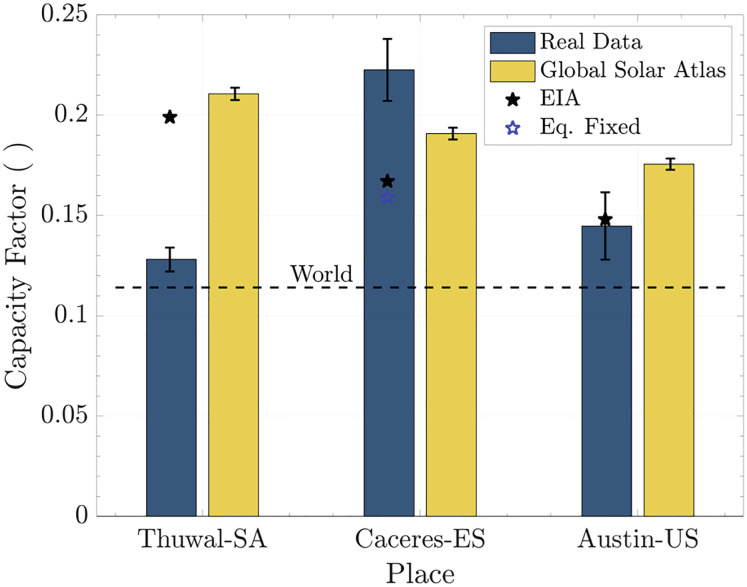
Real performance of solar arrays. The black color is data from EIA (53); the stars are the mean for the country, in which the array is placed, and the dashed line is the world mean. The yellow bars are reference data based on the Global Solar Atlas (52). The blue bars are the actual data from solar PV arrays. The solar array in Càceres has a double-axis solar tracking system, and the blue star represents its performance without tracking and at the optimum tilt angle.

Fig. 7 shows the three locations investigated by us. The yellow bars are the estimated CFs based on the Global Solar Atlas model that accounts for the effects of location, solar radiation, and air temperature (52). The blue bars are the actual data from three solar PV arrays. The solar array in Càceres, Spain, has a double-axis solar tracking system, and the blue star represents the panel’s performance if we assume that the array was fixed and at the optimum tilt angle. The black color encodes the data from EIA (53), which also represent the actual reported data. The stars represent the mean of the respective country where the array is placed, and the dashed line refers to the current global mean. Detailed performance of these arrays is discussed in *SI Appendix*, section 9.

Thuwal, Saudi Arabia, is an illustration of expected and real performance in a dusty environment. The real performance is 60 of the estimate by the Global Solar Atlas. This difference is due mainly to dust deposition; the recent measurements indicate average deposition of 11month (54). The current fortnightly cleaning with fresh water seems to be insufficient to attain the expected performance.

Overall, the Global Solar Atlas overestimates array performance (simpler models will yield even worse estimates) because it neglects the impact of dust. Also, the surface air temperature should be replaced by panel temperature, which requires a more sophisticated model. For example, when comparing the actual performance of the arrays with an expected performance estimated from an average of the local incident radiation, Austin delivers 64%, Càceres 82%, and Thuwal 48% of the expected power output (*SI Appendix*, section 9). In summary, the simpler the approach, the higher the deviation from reality is.

The real data measured and reported as the blue bars can be used as a reference. Under similar conditions, the performance of a similar array will not change drastically. The most important observation is that ambient conditions interfere much more than is commonly reported; the combo of high cell temperature and dust could undermine the future PV projects that will take place in the hot and arid regions of the world.

## Conclusions

Faced with the urgency of decarbonization and to avoid the harsh impacts of climate change, researchers have adopted the concept of CF, which is a straightforward, consistent indicator of the efficacy of diverse energy systems. CF is objective and informs about the average performance of a specific electricity generation technology. Real data must be used to estimate capacity factors to avoid wishful thinking and overestimates.

The capacity requirements for the replacement of fossil fuels or expanding the current energy system provide a clear insight into the scope of the challenge. Practically doubling the current energy system in place poses hidden infrastructural challenges that are not obvious and commonly neglected.

Current models and projections for solar photovoltaic power generation overestimate its average power output. We need more refined models that account for ambient conditions, such as panel temperature and dust deposition. Some approaches are close to the back-of-the-envelope calculations, which provide misleading information that may put the net-zero agenda off-track.

## Materials and Methods

CF was calculated as

[1] $$\begin{array}{c} \mathrm{CF}=\frac{\mathrm{Power}\;\mathrm{Produced}}{\mathrm{Maximum}\;\mathrm{Possible}\;\mathrm{Power}\;\mathrm{Output}}. \end{array}$$

The power output refers to the energy produced over the period analyzed. The maximum possible power output is delivered when the device or system analyzed functions uninterruptedly over a defined period. In our case, the reference period is 1 y.

We are calculating the CF based on the information available in the EIA (53) database, which can be expressed as

[2] $$\begin{array}{c} \mathrm{CF}=\frac{{\mathrm{Electricity}}_{\mathrm{Gen}.}}{{\mathrm{Electricity}}_{\mathrm{Cap}.}\times 8760}. \end{array}$$

The Electricity_Gen._ is the equivalent of net electricity generation (in Wh) that excludes the energy consumed by the power plant. The Electricity_Cap._ corresponds to the nominal capacity installed (in W) in a given year, and 8,760 corresponds to the number of hours in a year.

The CF are calculated by source (i.e., biomass fuels, fossil fuels, geothermal heat, water, uranium, solar PV, and wind) for each country and the world. Subsequently, the mean CF is calculated over the 2000 to 2017 period. In order to have a better perspective of how CFs are evolving, the regional calculations were made. The regions are the following: Africa (excluding north), Asia, Commonwealth of Independent States (CIS), Europe, Latin America, Middle-East and North Africa (MENA), North America, and Oceania. A detailed list of the countries belonging to each group is available in the Supplementary Information (*SI Appendix*, Fig. S6).

In addition, a weighted CF (CF_W_) was calculated,

[3] $$\begin{array}{c} {\mathrm{CF}}_{W}=\sum_{n}CF_{n}\times W_{n}. \end{array}$$

The summation is over countries to estimate the regional weighted CF. For the world, the summation is over regions.

The weight consists of the electricity share generated by a specific country or region, which is case-dependent and can be expressed as

$$W=\left\{\begin{array}{c} \frac{{\mathrm{Electricity}}_{\mathrm{Gen}.}\;\mathrm{Region}}{{\mathrm{Electricity}}_{\mathrm{Gen}.}\;\mathrm{World}},\;\mathrm{for}\;\mathrm{World}\;{\mathrm{CF}}_{W},\\ \frac{{\mathrm{Electricity}}_{\mathrm{Gen}.}\;\mathrm{Country}}{{\mathrm{Electricity}}_{\mathrm{Gen}.}\;\mathrm{Region}},\;\mathrm{for}\;\mathrm{Regional}\;{\mathrm{CF}}_{W}. \end{array}\right)$$

CFs greater or equal to 1, equal to 0, or ∞ were removed from the calculations. Also, when one of the previous conditions was identified, the compromised data (i.e., electricity generation) related to that condition were removed.

For the solar section, the EIA data are based in the procedure reported above. The Global Solar Atlas data were collected from their website for the city where the array is located (52). The real data reported are the average over the total period with access to the data, based on an annual mean and SD. The detailed data are available in *SI Appendix*, section 9.

## Supplementary Material

Appendix 01 (PDF)

## Data Availability

All algorithms developed by the authors. Data were collected from previously specified sources and posted on Zenodo (https://zenodo.org/record/6565027#.Yx9kHnbMI2w).
