# Supplementary material for: Capacity factors for electrical power generation from renewable and nonrenewable sources
Source: Proc Natl Acad Sci U S A. 2022 Dec 20;119(52):e2205429119. doi: 10.1073/pnas.2205429119 (PMC9907140; doi:10.1073/pnas.2205429119)
Supplement: Supplementary file 1 — Appendix 01 (PDF) [file pnas.2205429119.sapp.pdf]

PNAS
www.pnas.org

Supplementary Information for

Capacity factors for electrical power generation from renewable and nonrenewable sources

Natanael Bolson, Pedro Prieto and Tadeusz Patzek

Corresponding Author: Tadeusz Patzek.
E-mail: tadeusz.patzek@kaust.edu.sa

This PDF file includes:

- Supplementary text
- Figs. S1 to S42 (not allowed for Brief Reports)
- Tables S1 to S2 (not allowed for Brief Reports)
- Legend for Dataset S1
- SI References

Other supplementary materials for this manuscript include the following:

- Dataset S1

Supporting Information Text

1. Background

We all must learn how to tell parallel, scientifically truthful stories about competitive and reliable electricity generation and transmission. Capacity factor is one such important story.

As Patzek's own work on large ecological systems and renewable energy supply(1–6), and a recent brilliant paper by Giampietro and Bukkens(7) explain, the emergent properties of a complex system cannot be predicted by analyzing its parts separately. Thus we know fully well that a quasi steady-state average model like capacity factor is but a small part of the full understanding of a Green Transition problem. A complex system cannot be fully represented by any one model in particular(8–10). Those wanting to use quantitative information for decision-making about complex issues therefore face the standard predicament identified by Box(11): "all models are wrong, but some are useful". This does not mean that scientific inquiry is useless. Even if "true models" do not exist (like the capacity factor model), we can still tell useful stories about complex problems by selecting different perceptions of the system that we judge useful for our purpose (*e.g.*, for guiding action)(12).

From this point of view, the capacity factor model we present in this paper is a useful story that informs decision makers about a key aspect of supplying uninterrupted electricity to end-users, and it cannot inform them about other aspects. Where we seem to differ with some economists is that we are engineers, and focus on one subset of the problems[1] we deem to be important, and economists seem to focus on another subset[2]. But this is not a mutually exclusive proposition. Our model shows a very useful comparison of all sources of electrical power around the world, allowing people to make direct comparisons, even if in a limited sense. The often unspoken and unreferenced differences among different tellers of the Green Transition

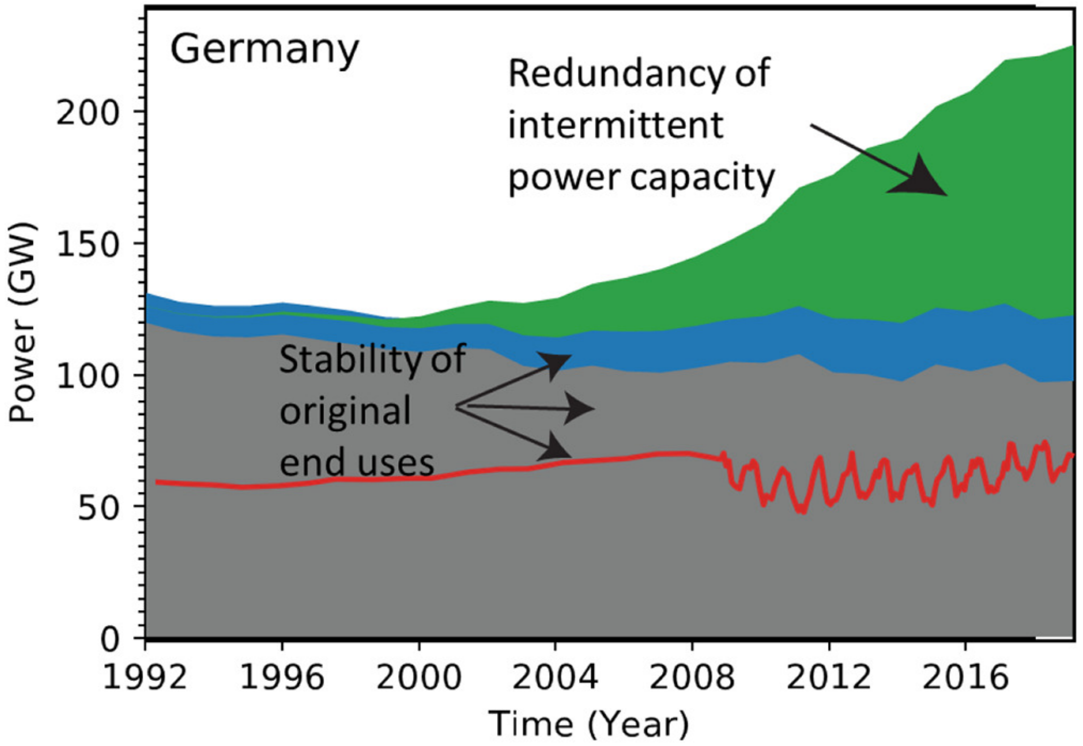

Fig. S1. The common belief that "when the cost of the kWh of alternative sources will be lower than that of the conventional sources, the conventional producers will be forced out of the market" is simply not true. Power capacity related to intermittent sources grew dramatically in Germany, but the size of power capacity related to conventional sources remained largely unaffected. Due to a lack of an adequate storage capacity, conventional sources are still needed to guarantee biophysical viability, see the Patzeks' story in Section 10. As a matter of fact, before the arrival of alternative electric sources, the cost of producing electricity with "baseload end-uses" and "peak end-uses" was different, and still the two types of end-uses (techniques of production) coexisted without there being an issue. The red line shows actual power generation. Fig. 4 and text in(7).

stories are in the treatment of renewables and how they inevitably must emerge. To shed some light on some of the many possible differences, let's quote from pages 8 and 9 in(7):

> The kWh paid by the consumers are "biophysically viable electricity", whereas the kWh sold by producers are mere inputs of electric charges that still must be blended and harmonized in the grid.
>
> Framing the issue in this way, we can immediately detect a systemic problem with the biophysical viability of the class of end-uses producing electricity from intermittent primary sources: they may produce electricity when

[1] Technical viability, uninterrupted power supply, efficiency,. . . , and economics far behind.

[2] Such as economics, efficacy, Levelized Cost of Energy (LCOE), replacement, etc.

it is not needed, and they may not produce electricity when it is needed. In other words, not all kWh generated are the same when considering biophysical and economic viability - they do not have the same value. There are storage solutions that can convert electricity into chemical energy such as batteries or hydrogen fuel, or into mechanical-gravitational energy such as pumped-hydro. However, *without the availability of large-scale storage capacity, intermittent end-uses do not have the same usefulness for running the grid as traditional end-uses* [Italics TWP].

Having stated the former, we can now clarify the confusion over different concepts essential to understanding the existing predicament experienced with the attempt of a large-scale integration of intermittent sources of electricity (solar and wind) into existing grids. We can distinguish between four factors: (1) the quantity of electric power that is distributed in the grid (produced and used at any moment), a concept associated with biophysical viability; (2) the mix of different end-uses expressed by those producing and using electricity (referring to different types of technology and social practices), a concept associated with economic viability. These end-uses can be changed by the market mechanism, but at a slow pace. Changes in patterns of production and consumption of electricity are not determined by changes in "consumer behavior" but by changes in social practices (such as Hoteit's parents adapting their lifestyles and electricity use to grid collapse in Lebanon, Section 10); (3) the amount of power capacity and infrastructures associated with the different end-uses supplying secondary energy carriers in the form of electric power; and (4) the amount of storage capacity available in the grid to compensate mismatches between supply and consumption. The latter factor is the *missing character in the existing storytelling about the decarbonization of electricity supply* [Italics, TWP]. Storage capacity can dramatically change the usefulness of intermittent sources by allowing to store electricity produced when it is not needed and release electricity when required in excess of the available supply. However, for the moment we do not have large scale solutions. . . .

. . .

In conclusion, the problem of a large-scale integration of intermittent sources of electricity in the existing typology of centralized grid lies with the lack of storage capacity and not with the "right" price of kWh.[Bold, TWP]

The last sentence in this quotation seems to be key in explaining the differences between our view of the "economics or efficacy of a project or the ability of a resource to effectively replace another" and these of several economists, see the important Figure S1.

As we stated in the paper, almost all practical analyses that compare different electrical power systems rely on LCOE. The most popular approach presented in Wikipedia belongs to Lazard, whose latest report, version 15.0 LCOE, https://www.lazard.com/-media/451905/lazards-levelized-cost-of-energy-version-150-vf.pdf, contains an explicit reference to the capacity factors they use to perform example calculations, also posted in Wikipedia:

"Solar projects assume illustrative capacity factors of 21% – 28% for the U.S., 26% - 30% for Australia, 26% - 28% for Brazil, 22% - 23% for India, 27% - 29% for South Africa, 16% - 18% for Japan and 13% – 16% for Europe."

Compared with our 15% average estimate for the US (the 27% outliers belonging to the expensive biaxial tracking systems in Guam, and 13-16% belonging to Patzek's excellent arrays in Austin, TX), Lazard's estimate of 21 - 28% is quite high. The same goes for Europe, which is firmly 11 - 12% for us and 13-16% for Lazard. Dr. Bolson's home country, Brazil, is 11% on the average, because of high humidity and clouds, compared with Lazard's 28%. India is 18% for us and 22 - 23% for Lazard, and so on. For Europe, which has the firmest most stable estimates, Lazard's numbers are 18-45% too high.

In particular, for the continental US, Lazard's numbers are 40-87% higher than average, and his calculated LCOEs are correspondingly lower. Then there is the problem of power storage. Solar PV arrays only work during sunny days. At a significant penetration of solar PV (and wind), massive storage systems are needed to provide the 24/7 biophysical electricity to customers, who in our judgement would have to power down at night.

A lithium ion battery backup system that would allow the Patzeks to go off-grid for 10 years, after significantly curtailing their current power use (85% AC), would cost at least as much as the arrays, doubling their electricity generation cost. This means that *if* there were a viable storage system, the Lazard's LCOE for solar PV could be up to $2 \times 2 = 4$ times higher for the continental US. Thus, as we show in Section 10, the biophysical price per kWh really does not matter when one compares the intermittent renewables with the 24/7 baseload and peaking plants (Figure S1). Where there is no grid, people are forced to make deep changes in their lifestyles and already live their own Green Transitions, see Section 10. Biophysically, 1 kWh of the *sometimes* electricity is qualitatively different from 1 kWh of the *always* electricity.

2. World Capacity Factor

Figure S2 is the box plot of the world's average capacity factors (CFs) and the weighted mean CFs of different electricity sources. The global capacity factors for the different sources have small variances over the observed period of 2000-2017. The CF variability can be substantial among regions or countries, see section 6 and 8.

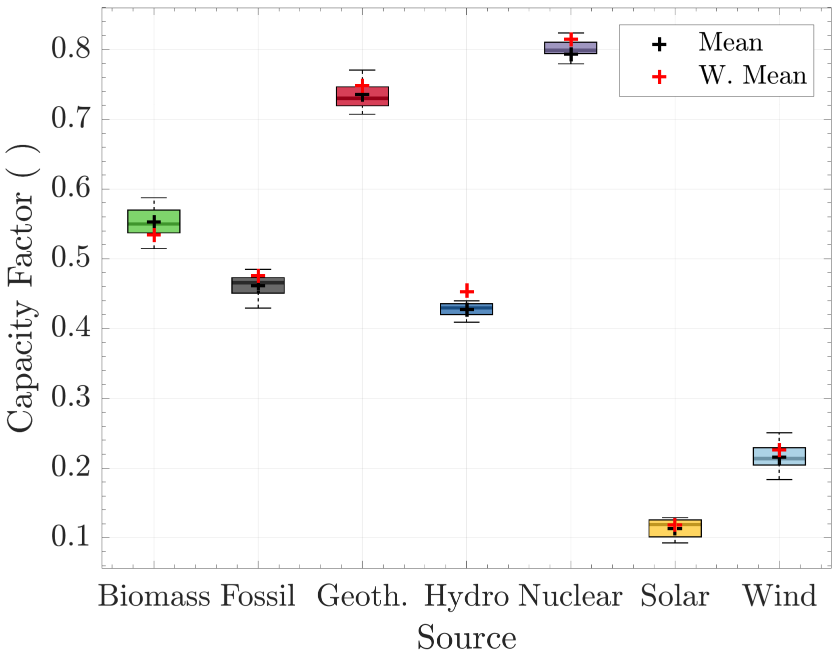

Fig. S2. World capacity factors for key electricity sources. The black cross is the mean; the black line in the middle of the box is the median, the upper limit of the box is the 75th quantile and the lower limit is the 25th quantile. The whiskers are $1.5\times$ interquantile range. The red cross is the weighted mean.

3. Fossil Fuel Electricity Sources in Detail

The EIA database(13) does not distinguish among the fossil sources of electricity. The shares of total electricity by source can be obtained from the World Bank database for coal, natural gas, and oil(14–16). The installed capacity is available through the EIA's International Energy Outlook(17–19). Figure S3 helps us to understand the big picture for the fossil fuel electricity generation worldwide. Before further discussion, it is important to clarify that each fossil source plays a different role. While coal generates baseload electricity, natural gas usually satisfies peak demand, and oil is used mostly for emergency needs or backup. Figure S3a shows the capacity factors (CFs) for the fossil sources. The observed values are typical of the roles these sources play in the electric power mix. Additionally, fig. S3b shows the shares of nominal capacity and electricity generated. Coal and natural gas have higher shares of the electricity generated, because of their roles in the grid. In contrast, oil has more idle capacity due to its role as a backup or emergency electricity source.

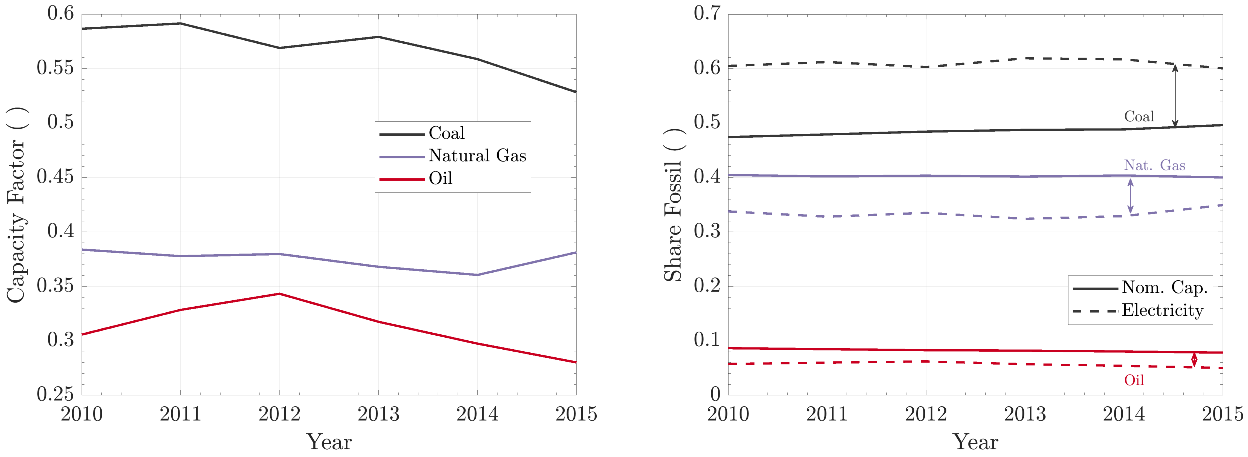

(a) (b)

Fig. S3. Recent history of fossil fuel electricity worldwide. a) Capacity factors; b) Shares of fossil electricity sources.

4. Capacity Factors of Fossil Fuels

The current decrease in the fossil fuel capacity factors could be associated with several interrelated causes. We explore two key hypotheses:

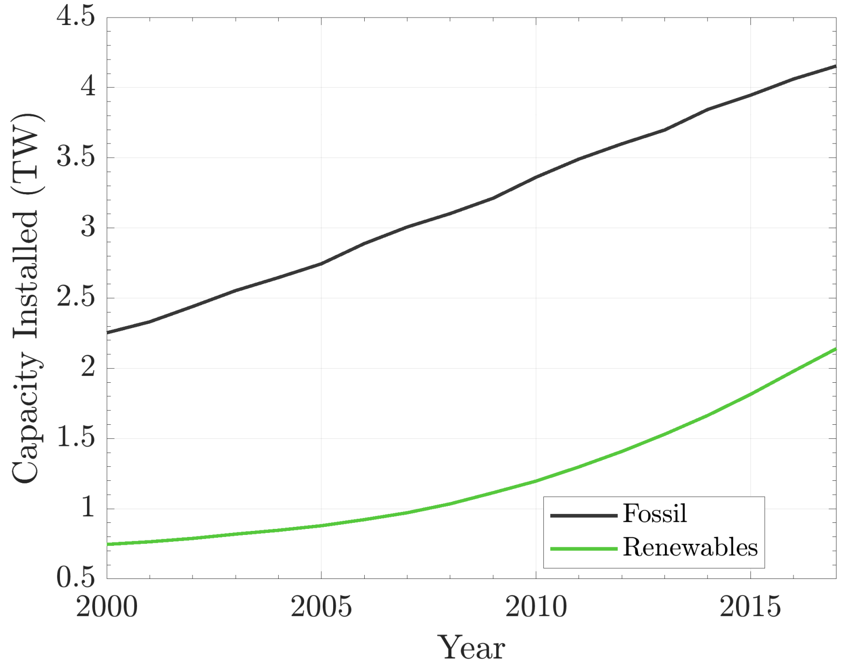

Fig. S4. Total installed capacity for fossil fuels, *i.e.*, coal, natural gas, and oil, and renewables, *i.e.*, biomass, geothermal, hydro, solar, and wind.

1. Replacement of fossil fuels by renewables

    This hypothesis is negated by the ongoing expansion of fossil fuel-based generation capacity; the installed capacity of renewables is growing at an even faster pace, see fig. S4. However, up until now, supply growth has been implemented as electric power additions to the existing supply mix, rather than proper replacement.

2. Overestimation of electricity demand

    This hypothesis reflects long construction times required to build a fossil power plant. To match predicted future demand, the decisions to build are made several years earlier. Therefore, a mismatch between the projected and current electricity demand may result in an increase of idle capacity, see fig. S5.

    Electricity generation and GDP follow similar growth trends, see fig. S5a, resulting in a strong linear correlation in fig. S5b. The average time to build a fossil power plant is five years(20), and a projected GDP growth is a good indicator of the corresponding increase in demand five years down the road. Figure S5c shows the mismatch of electricity generated based on the forecasted and real GDP growth. We assume that renewable and nuclear power plants will continue to operate as before, and that the fossil fuel-based generation will absorb demand fluctuations. The impact of the mismatch on CF is shown in fig. S5d. As we can observe in the forecasted scenario, the capacity factors are fixed at the 2008 levels, before the economic crisis. A more illustrative depiction of the overestimation mismatch is shown as the shaded areas in fig. S5c and fig. S5d. The blue shaded area is the total overestimation. The green shaded area is a safety factor, a small overestimate that could occur due to uncertainties. The red shaded area is the difference of blue minus green, or the significant difference between the predicted and actual electricity consumption.

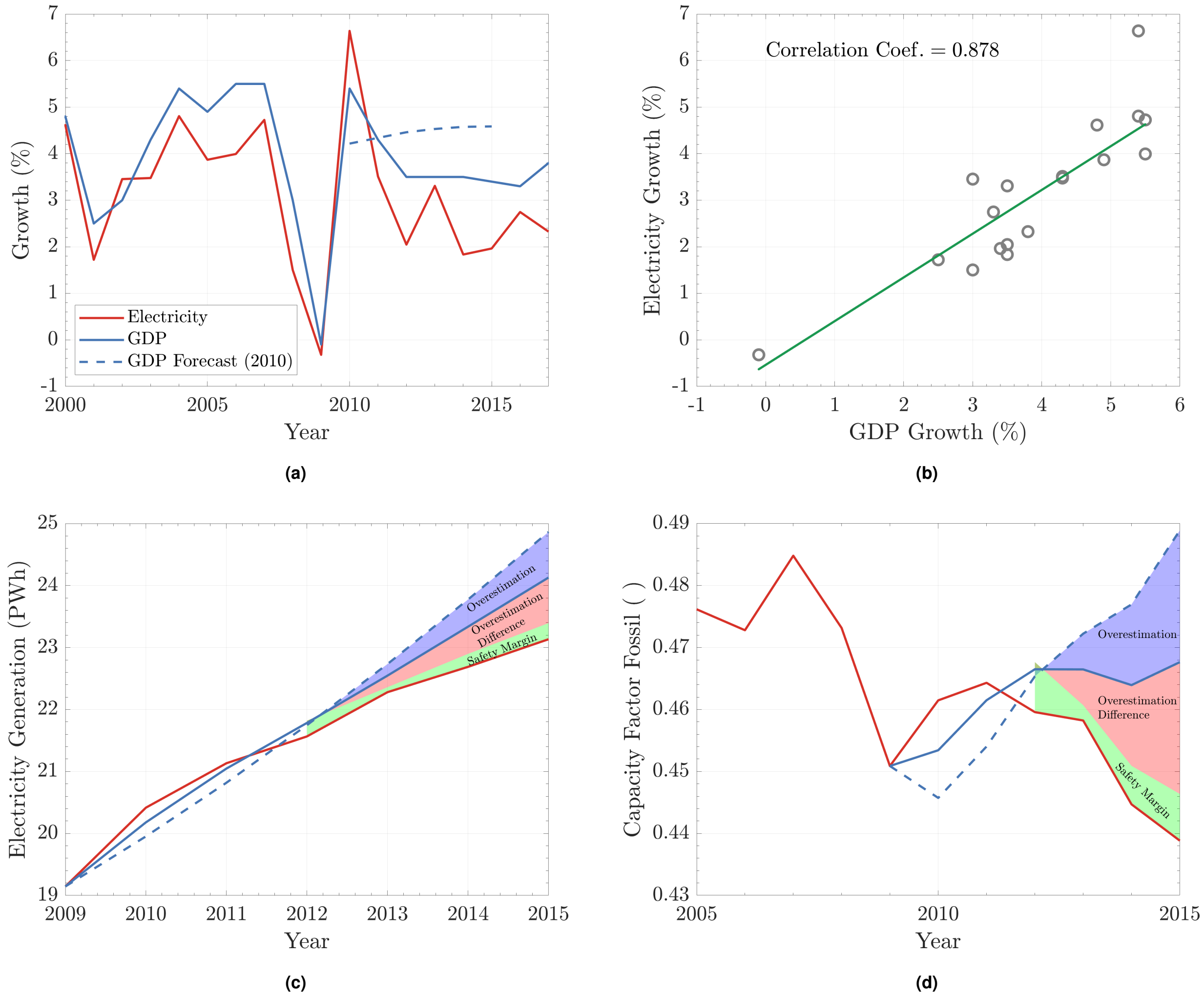

Fig. S5. Factors affecting fossil fuel CF. The red line represents the actual electricity generated(13); the solid blue line represents the GDP growth by IMF(21), and the blue dashed line represents the forecast of GDP made in April 2010 by IMF(22). a) Growth of electricity generated and GDP. b) Correlation between electricity growth and GDP growth. c) Electricity generated, the red line is historic data, the solid line is a projection based on real GDP growth, the dashed blue line is electricity generation based on the forecasted GDP. d) CF of fossil fuel sources; the red line denotes real values, the solid blue line is based on GDP growth, and the dashed blue line based on forecasted GDP. The blue shaded area represents the overestimate. The red shaded area is the mirrored values of the blue area, which illustrates the difference between the real GDP and current electricity values. The green area is difference remaining even if the overestimate is removed; in practice, it is a safety margin due to uncertainties.

5. Regional Division

The following regional division was adopted

- **Africa**

  Angola, Benin, Botswana, Burkina Faso, Burundi, Cabo Verde, Cameroon, Central African Republic, Chad, Comoros, Congo-Brazzaville, Congo-Kinshasa, Cǒte d'Ivoire, Djibouti, Equatorial Guinea, Eritrea, Eswatini, Ethiopia, Gabon, Gambia, Ghana, Guinea, Guinea-Bissau, Kenya, Lesotho, Liberia, Madagascar, Malawi, Mali, Mauritania, Mauritius, Mozambique, Namibia, Niger, Nigeria, Reunion, Rwanda, Saint Helena, Sao Tome and Principe, Senegal, Seychelles, Sierra Leone, Somalia, South Africa, South Sudan, Sudan, Tanzania, Togo, Uganda, Western Sahara, Zambia, and Zimbabwe.

- **Asia**

  Afghanistan, Bangladesh, Bhutan, Brunei, Burma, Cambodia, Cayman Islands, China, Faroe Islands, Hong Kong, India, Indonesia, Japan, Laos, Macau, Malaysia, Maldives, Mongolia, Nepal, North Korea, Pakistan, Philippines, Singapore, South Korea, Sri Lanka, Taiwan, Thailand, Timor-Leste, Turkey, and Vietnam.

- **Common Wealth Independent States (CIS)**

  Armenia, Azerbaijan, Belarus, Georgia, Kazakhstan, Kyrgyzstan, Moldova, Russia, Tajikistan, Turkmenistan, Ukraine, and Uzbekistan.

- **Europe**

  Albania, Austria, Belgium, Bosnia and Herzegovina, Bulgaria, Croatia, Cyprus, Czech Republic, Denmark, Estonia, Finland, France, Germany, Gibraltar, Greece, Greenland, Hungary, Iceland, Ireland, Italy, Kosovo, Latvia, Lithuania, Luxembourg, Malta, Montenegro, Netherlands, North Macedonia, Norway, Poland, Portugal, Romania, Serbia, Slovakia, Slovenia, Spain, Sweden, Switzerland, and United Kingdom.

- **Latin America**

  Antigua and Barbuda, Argentina, Aruba, Barbados, Belize, Bermuda, Bolivia, Brazil, Chile, Colombia, Costa Rica, Cuba, Dominica, Dominican Republic, Ecuador, El Salvador, Falkland Islands, French Guiana, Grenada, Guadeloupe, Guatemala, Guyana, Haiti, Honduras, Jamaica, Martinique, Mexico, Montserrat, Nicaragua, Panama, Paraguay, Peru, Puerto Rico, Saint Kitts and Nevis, Saint Lucia, Saint Vincent/Grenadines, Suriname, The Bahamas, Trinidad and Tobago, Turks and Caicos Islands, Uruguay, and Venezuela.

- **Middle-East and North Africa (MENA)**

  Algeria, Bahrain, Egypt, Iran, Iraq, Israel, Jordan, Kuwait, Lebanon, Libya, Morocco, Oman, Palestinian Territories, Qatar, Saudi Arabia, Syria, Tunisia, United Arab Emirates, and Yemen.

- **North America**

  Canada, Saint Pierre and Miquelon, U.S. Pacific Islands, U.S. Territories, U.S. Virgin Islands, and United States.

- **Oceania**

  American Samoa, Australia, British Virgin Islands, Cook Islands, Fiji, French Polynesia, Guam, Kiribati, Micronesia, Nauru, New Caledonia, New Zealand, Niue, Northern Mariana Islands, Papua New Guinea, Samoa, Solomon Islands, Tonga, Tuvalu, Vanuatu, and Wake Island.

- **Antarctica**

Figure S6 illustrates the regional division described.

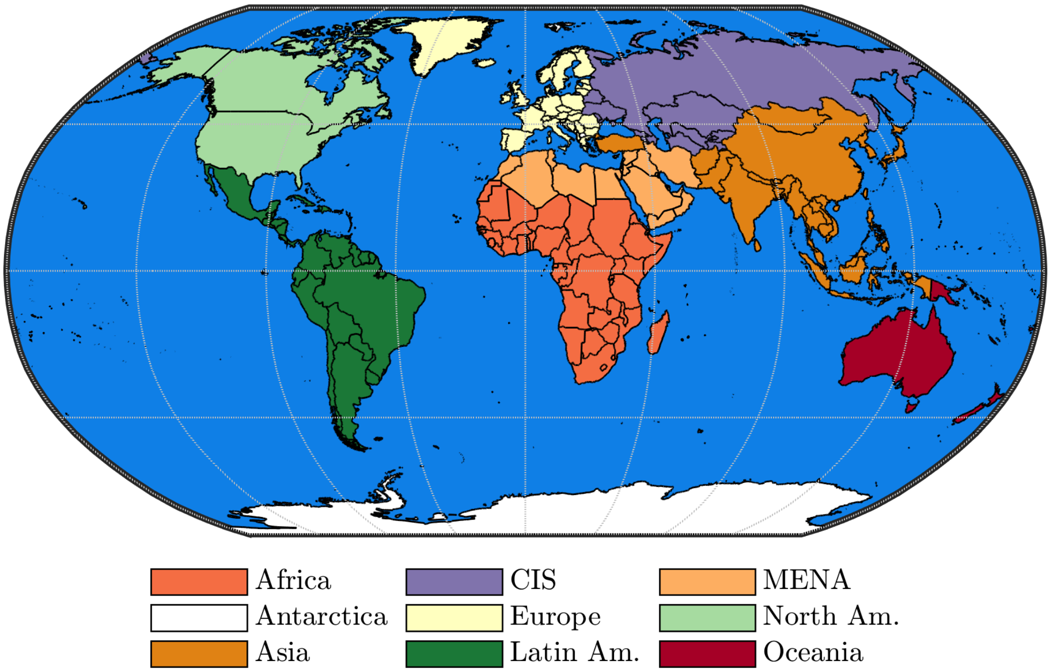

Fig. S6. Political division. CIS is the Commonwealth of Independent States, MENA is the Middle-East and North Africa. The country boundaries are plotted using ref. (23).

6. Capacity Factor by Region

Figure S7 shows a compilation of CFs by region. It allows a quick comparison of the suitability and/or importance of each electricity source for the specific region. Each box plot summarizes data scatter. The weighted mean gives an insight into the big electricity producers in each region and for all sources.

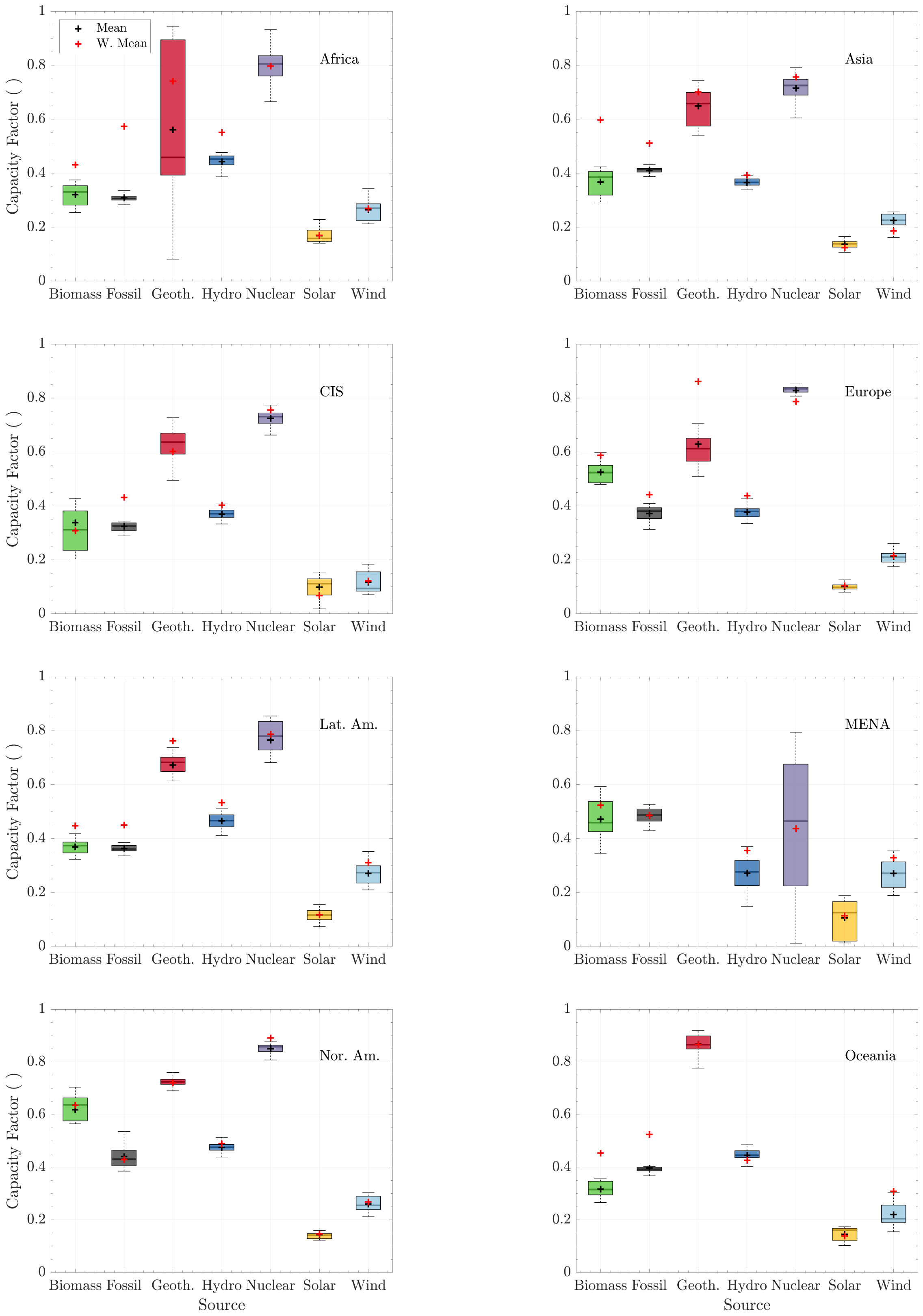

Fig. S7. Summary of CF by region. The upper limit of the box is the 75th quantile, the lower limit is the 25th; the whiskers are $1.5\times$ interquantile range. The black cross is the mean, and the red cross is the weighted mean.

7. Capacity Factor by Source

A. Biomass. Figure S8 displays the information used to calculate the biomass CF. The historical data showed in fig. S8a indicate the predominant biomass electricity producing region, Europe. In addition, fig. S8b shows the installed capacity that allows us to estimate the magnitude of electricity generated by biomass fuels. Also, it allows us to draw a comparison with fig. S8a, which shows that while North America reduced its share of total electricity generated from biomass, the total capacity installed grew over the investigated period.

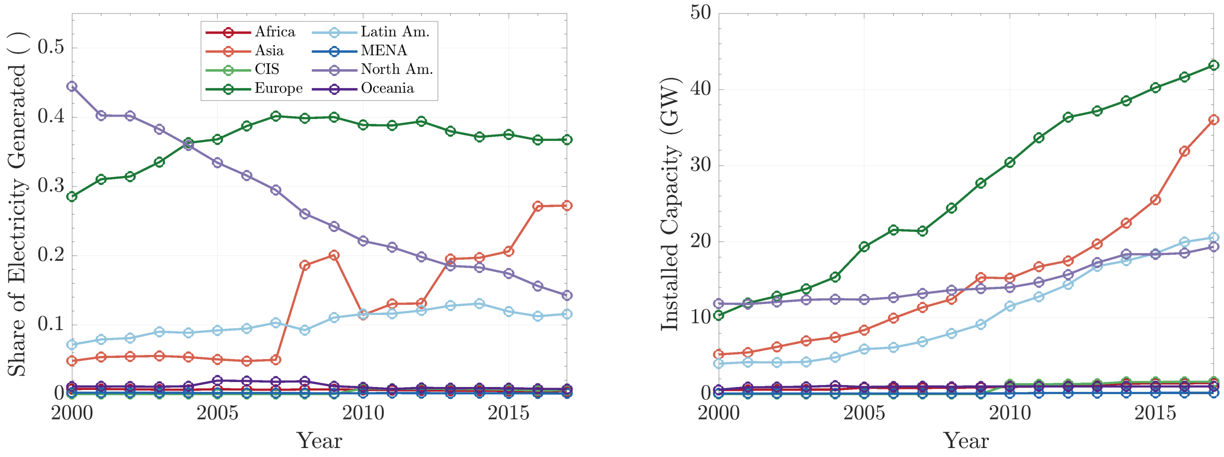

(a) (b)

Fig. S8. Biomass. a) Electricity generation share from biomass for the regions in fig. S6. b) Nominal installed capacity. Data from EIA(13).

Figure S9 shows the average CF and the weighted mean CF. It shows the distribution of capacity factors, and the weighted mean illustrates performance of big producers. The discrepancy between the weighted mean and mean implies that some countries in a given region have a strong reliance on biomass.

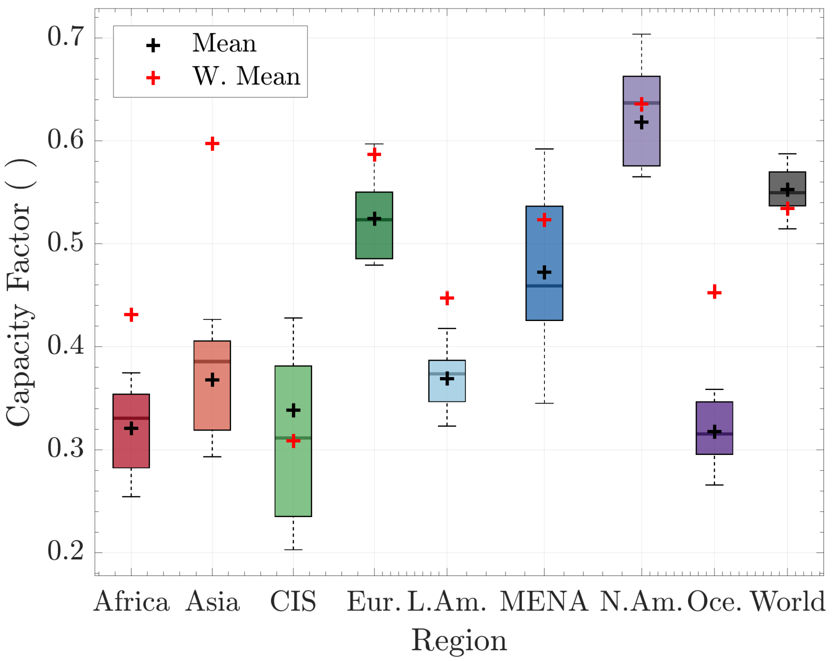

Fig. S9. Average biomass CFs for the regions in fig. S6.

Figure S10 shows the capacity factors' historical values and weighted capacity factor. While fig. S9 shows the average values, fig. S10 displays the full data, allowing us to observe the dynamic evolution. It indicates whether a given region is adopting

or ditching the biomass technology. The missing points in the CIS region are due to low data quality, which necessitated elimination of some data points.

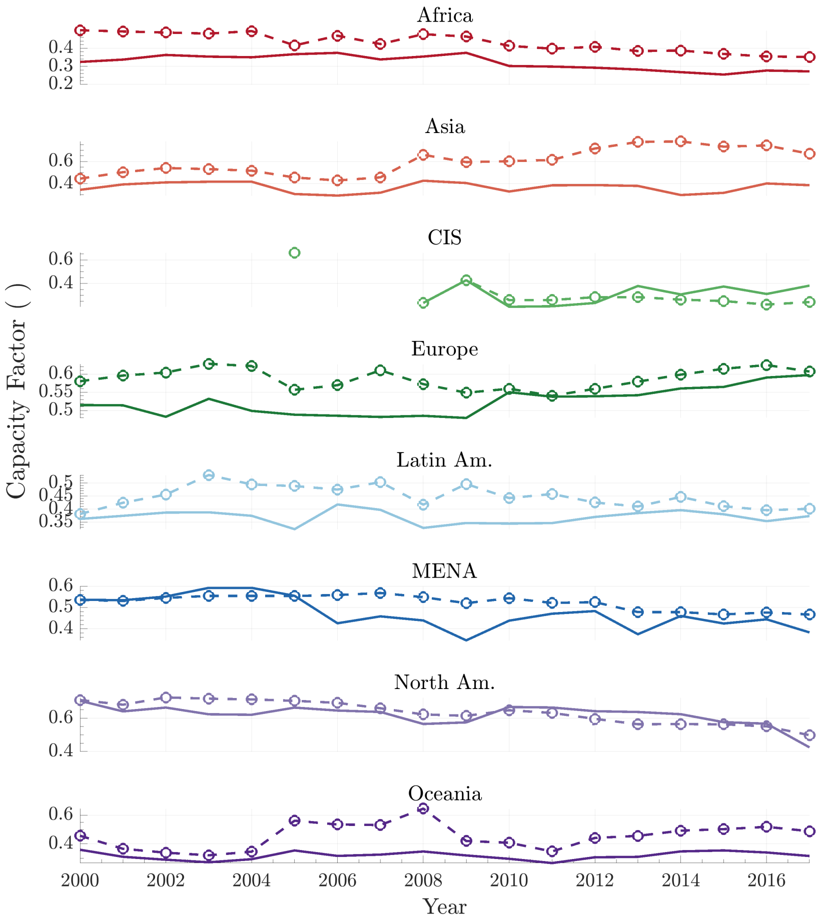

Fig. S10. Historical values for biomass CF.

B. Fossil Fuels. Figure S11 displays the information used to calculate CF. The historical data showed in fig. S11a highlight the predominant fossil electricity producer region, Asia. In addition, fig. S11b shows the installed capacity that allows us to estimate the magnitude of electricity generated by fossil fuels. A comparison of the trend shows that a significant growth of installed capacity occurred in Asia and at a lower scale in MENA, while growth in other regions was either negative or next to none.

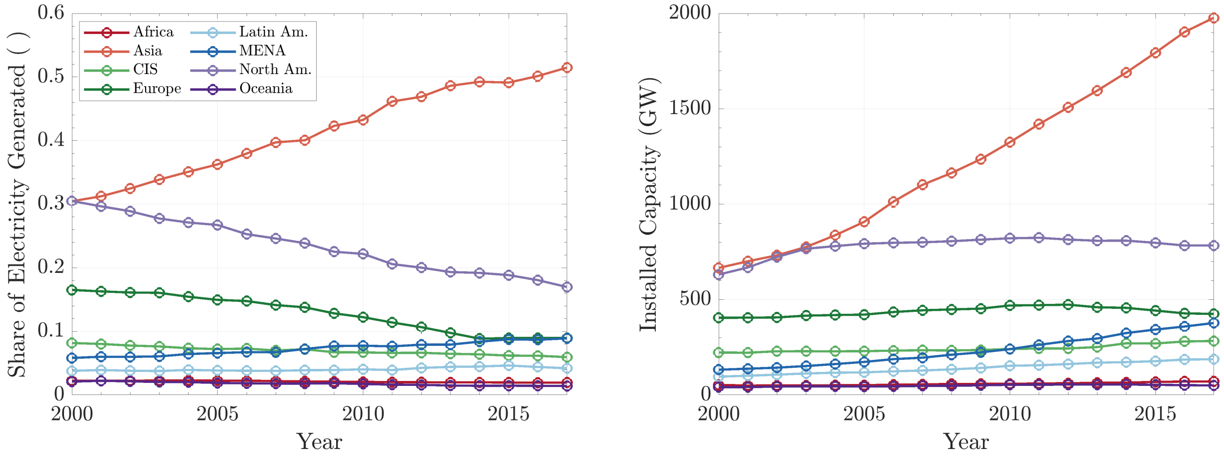

(a) (b)

Fig. S11. Fossil. a) Electricity generation share from fossil by the different regions; b) Nominal installed capacity. Data from EIA(13).

Figure S12 shows the average capacity factor and the weighted mean capacity factor. It shows the distribution of capacity factors, and the weighted mean illustrates performance of big producers. With the exception of MENA, all the other regions have a discrepancy between the average values and weighted values. It is a consequence of the contribution of big producers. In the case of Oceania, it reflects Australia's capacity factor and China's dominance in Asia. These discrepancies can also be due to the impact of more than one specific country with a strong reliance on fossil fuels.

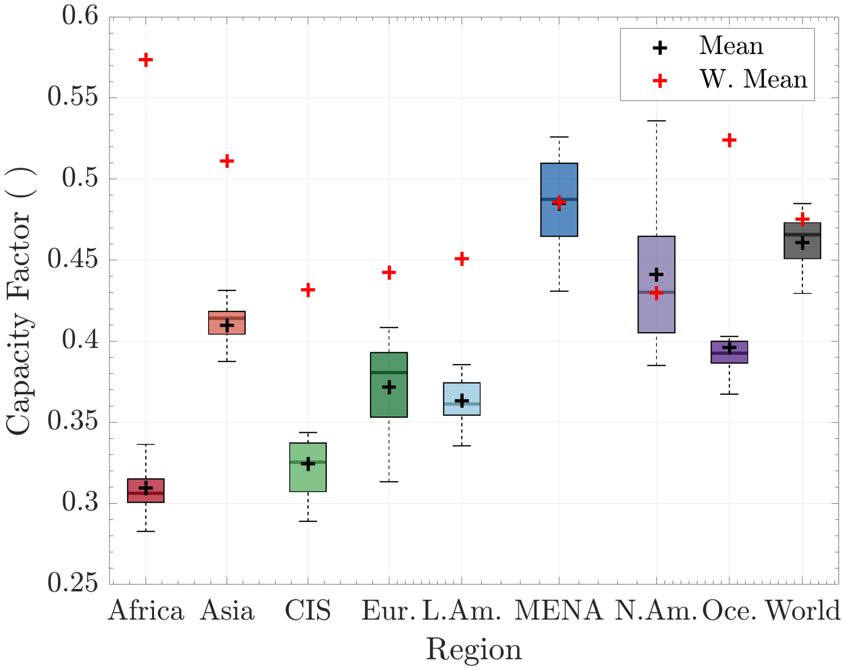

Fig. S12. Average values of fossil CF for the regions in fig. S6.

Figure S13 shows the historical values of the CFs and weighted CF. While fig. S12 shows the average values, fig. S13 presents the full data, allowing us to observe the dynamic evolution of CFs. In the MENA region, the weighted mean and mean have

194 similar values, indicating homogeneity around the region regarding reliance on fossil fuels.

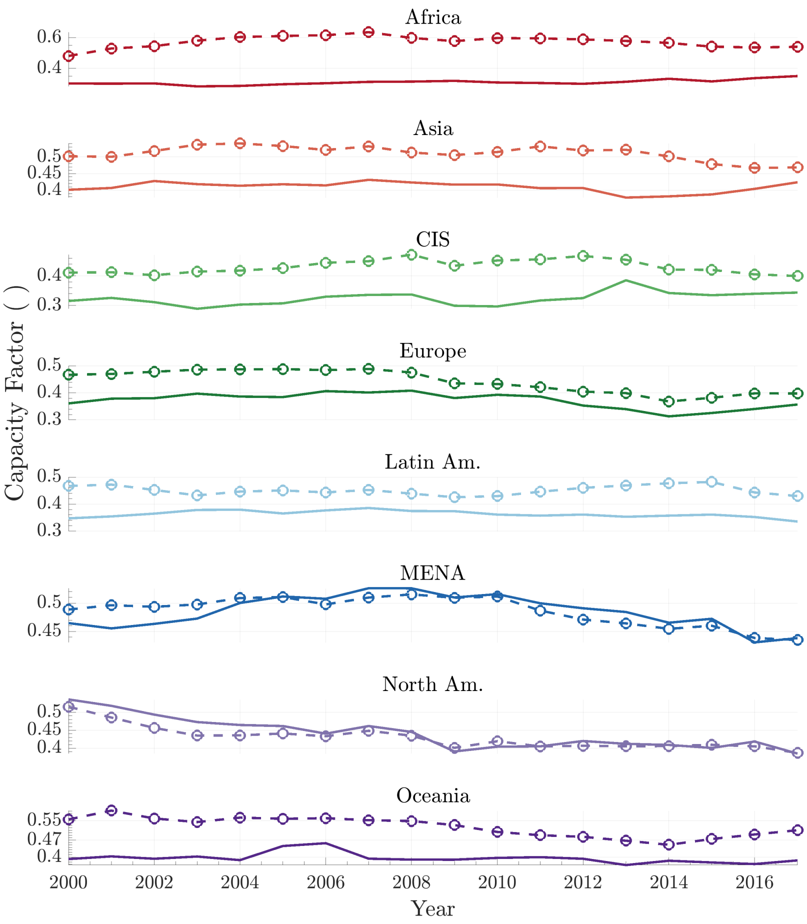

Fig. S13. Historical values for the fossil fuel CF.

C. Geothermal. Figure S14 displays the information used to calculate the CF. The historical data showed in fig. S14a indicate the predominant geothermal electricity producing region, Asia. In addition, fig. S14b shows the installed capacity that allows us to estimate the magnitude of electricity generated by the geothermal sources. The geothermal energy mix seems stable over the analyzed period; only Africa, Asia, and Oceania had a more significant expansion of installed capacity.

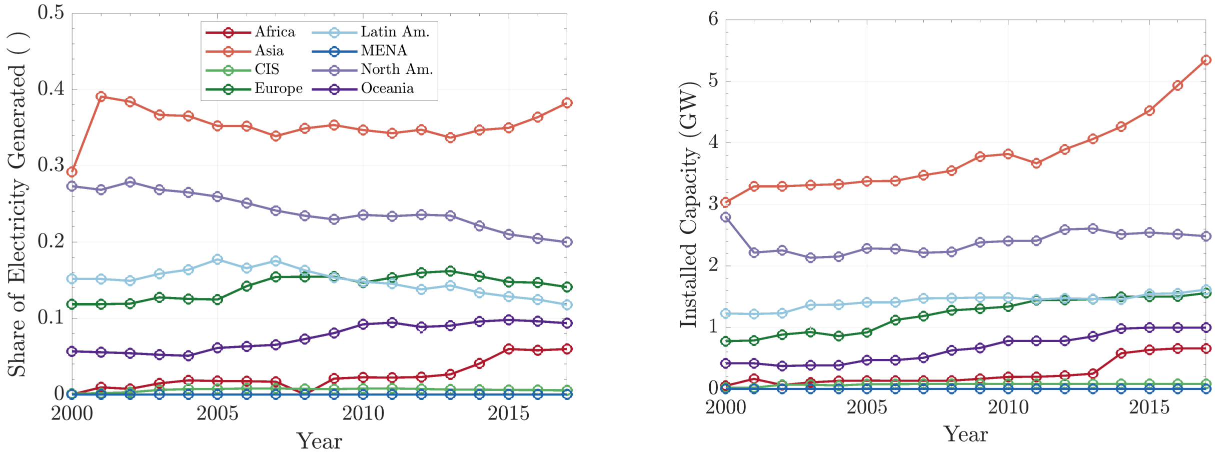

(a) (b)

Fig. S14. Geothermal. a) Electricity generation share from geothermal for the regions n in fig. S6. b) Nominal installed capacity. Data from EIA(13).

Figure S15 shows the average CF and the weighted mean CF. It shows the distribution of capacity factors, and the weighted mean illustrates performance of big producers. Africa has only two countries with geothermal, Ethiopia and Kenya. Ethiopia's production has issues. In some years, the capacity factor is near zero, it is not clear if it is because of the missing data or challenges faced at the two geothermal power plants.

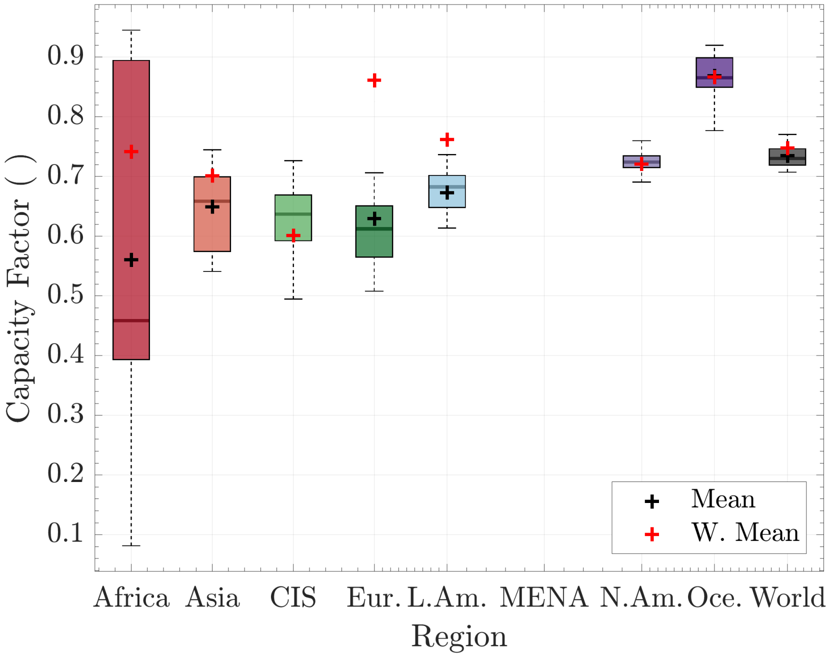

Fig. S15. Average values of geothermal capacity factor for the regions in fig. S6.

Figure S16 shows the historical values of the capacity factors and weighted capacity factor. While fig. S15 shows the average values, fig. S16 presents the full data, allowing us to observe the dynamic evolution. Here, when the mean and weighted mean overlap, in North America, the only country responsible for the overlap is the United States, and only Russia has geothermal in the CIS region.

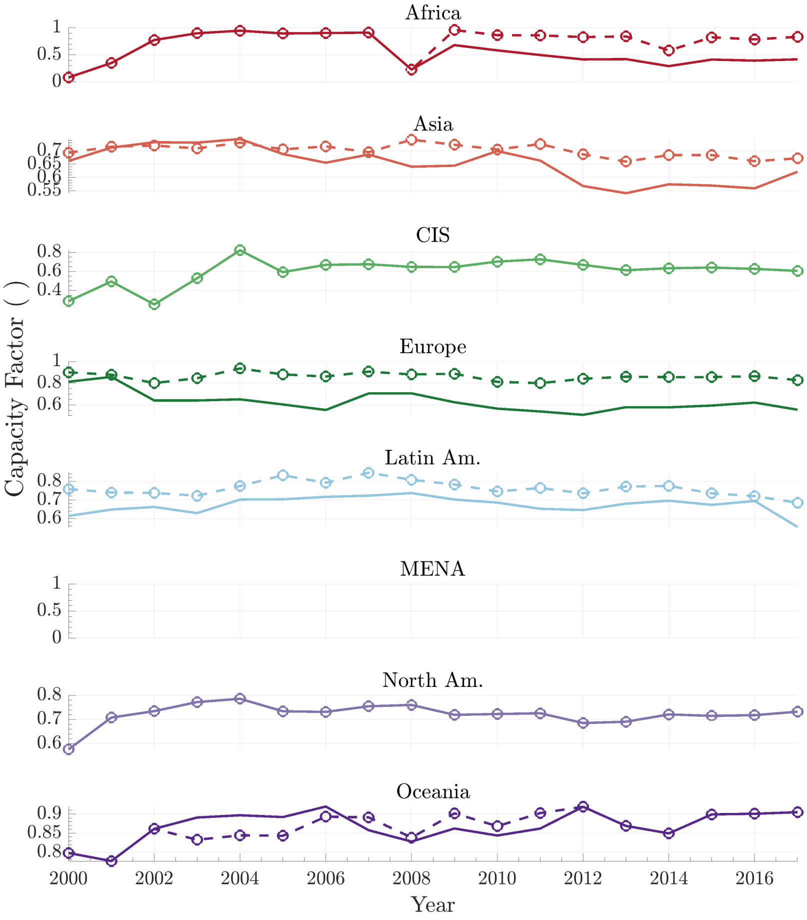

Fig. S16. Historical values for geothermal capacity factor.

D. Hydro. Figure S17 shows the information used to calculate CF. The historical data showed in fig. S17a show the predominant hydroelectricity producing region, Asia. In addition, fig. S17b shows the installed capacity that allows us to estimate the magnitude of electricity generated by water. Again, we can observe that Asia increased its installed capacity, consequently increasing the electricity generated from hydropower, while the other regions were practically constant except Latin America that saw a slight increase.

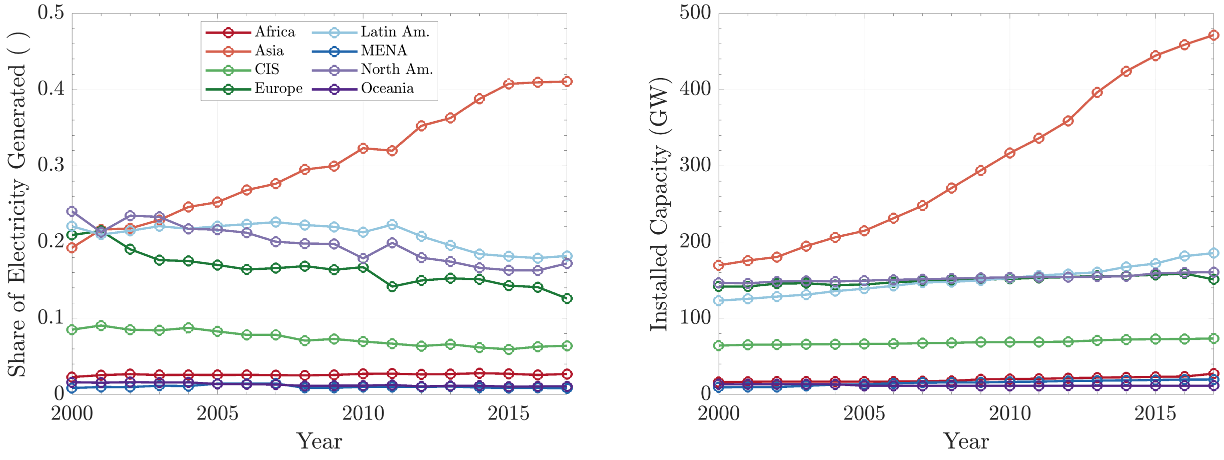

(a) (b)

Fig. S17. Hydro. a) Electricity generation share from hydro for the regions in fig. S6. b) Nominal installed capacity. Data from EIA(13).

Figure S18 shows the average CF and the weighted mean CF. It shows the distribution of capacity factors, and the weighted mean illustrates performance of big producers. Interesting observations can be made. In Latin America, the weighted capacity factor is dominated by Brazil, which is responsible for half of the hydroelectricity generated in the region. Africa has a handful of countries, which are big producers that unbalance the weighted mean. In MENA, a considerable discrepancy between countries with capacity factor near zero could be a consequence of political problems, and lack of river and lakes.

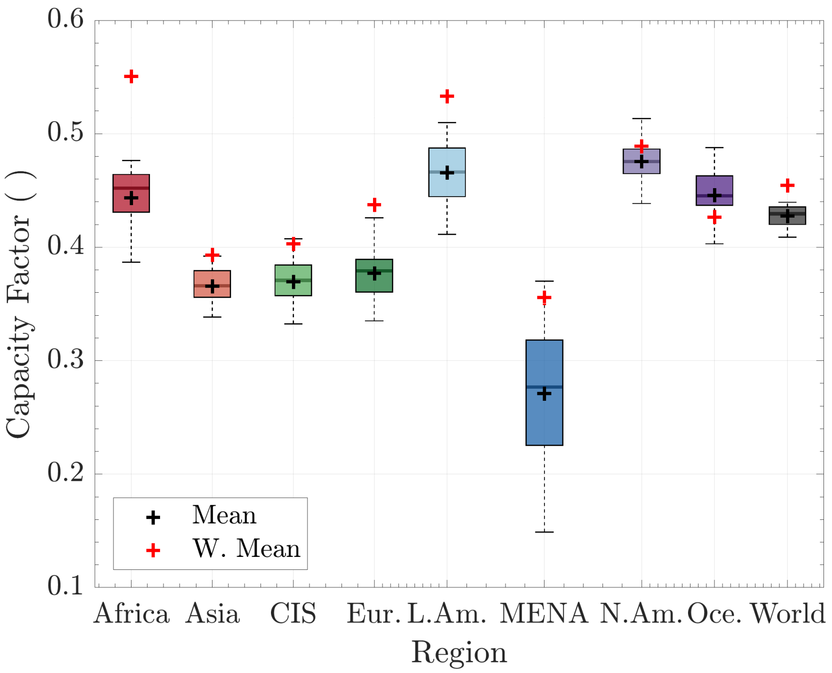

Fig. S18. Average values of hydro capacity factor for the regions in fig. S6.

Figure S19 shows the historical values of the CFs and weighted CF. While fig. S18 shows the average values, fig. S19 presents

the full data, allowing us to observe the dynamic evolution. An interesting observation in Latin America is the downward trend of the capacity factor, which could be a consequence of the hydro-climate extremes that have intensified since the 1950s.

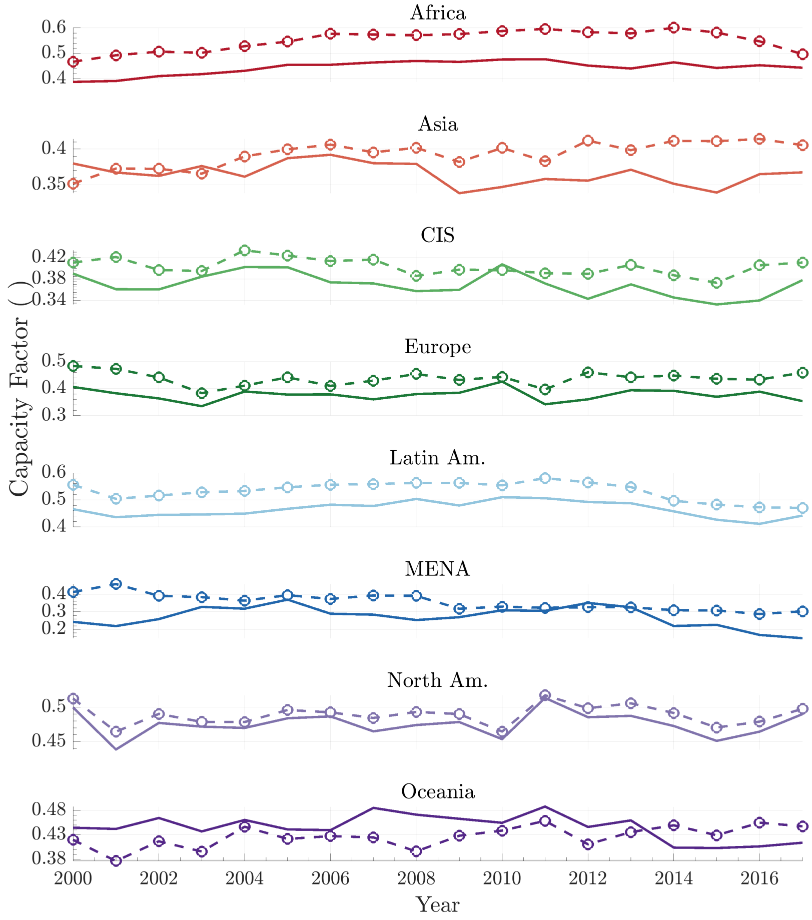

Fig. S19. Historical values for hydro capacity factor.

E. Nuclear. Figure S20 shows the information used to calculate capacity factor. The historical data showed in fig. S20a exhibit the predominant nuclear electricity producing regions, North America and Europe. In addition, fig. S20b shows the installed capacity that allows us to estimate the magnitude of electricity generated by uranium. Overall, the installed capacity of nuclear power plants has been steady. The decrease of installed capacity in Asia is due to the complete shut down of nuclear power plants in Japan after Fukushima's incident(24, 25). Europe has been implementing a nuclear power plant phase-out, and the reduction observed there is due to the shutdown of nuclear reactors in Germany.

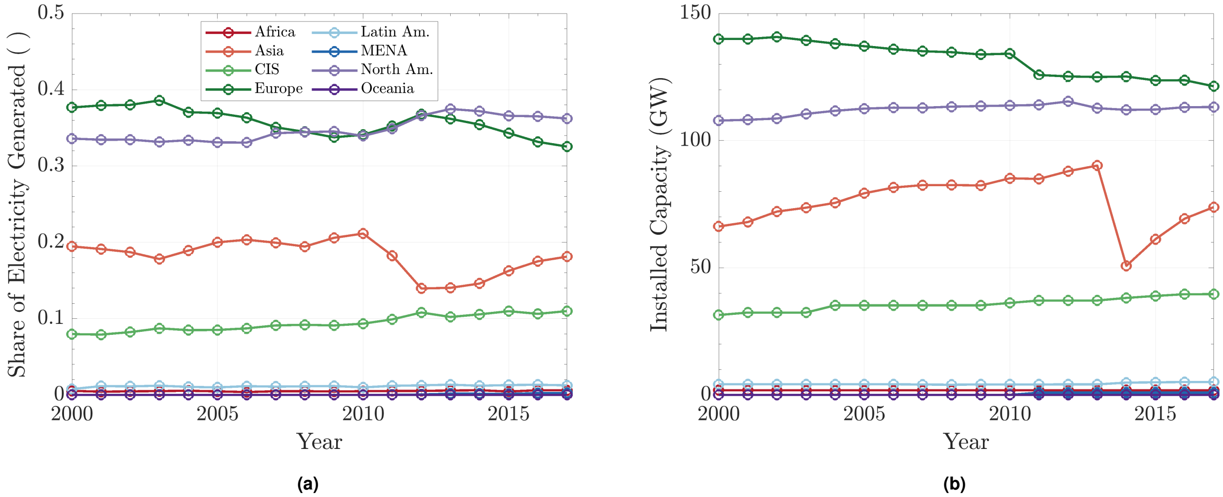

Fig. S20. Nuclear. a) Electricity generation share from nuclear for the regions in fig. S6. b) Nominal installed capacity. Data from EIA(13).

Figure S21 shows the average CF and the weighted mean CF. Overall, nuclear power has a small variance of its CF. The MENA region is an exception. Our database includes only Iran, starting in 2011, and with a low CF resulting from pilot plants and learning curve. In the most recent years, the CF reported is within a typical range of 0.8. We should also expect an expansion of nuclear capacity in MENA, driven by the Gulf states(26, 27).

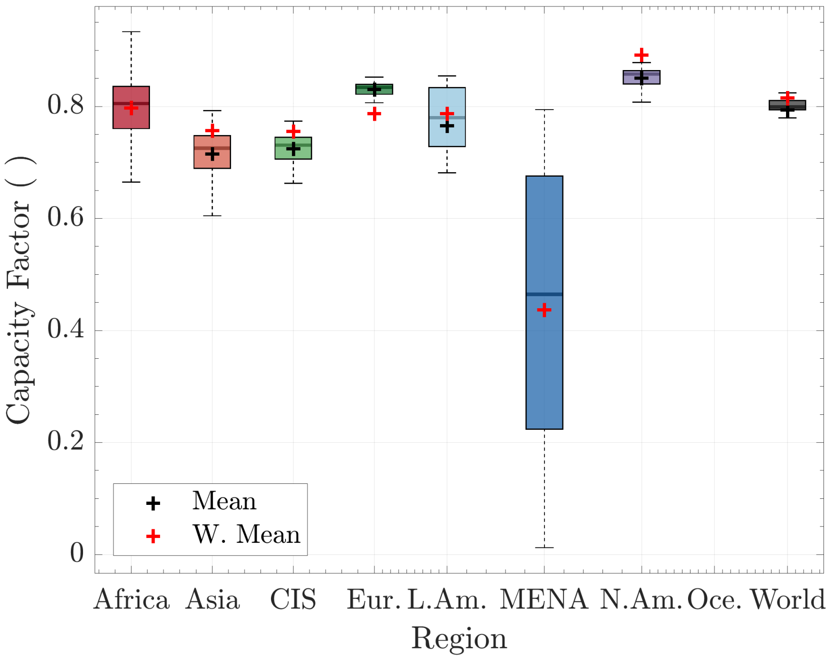

Fig. S21. Average values of nuclear capacity factor for the regions in fig. S6.

Figure S22 shows the historical values of the CFs and weighted CF. While fig. S21 shows the average values, fig. S22 presents

231 the full data, allowing us to observe the dynamic evolution. In the case of MENA, it reflects Iran's progress in nuclear power generation.

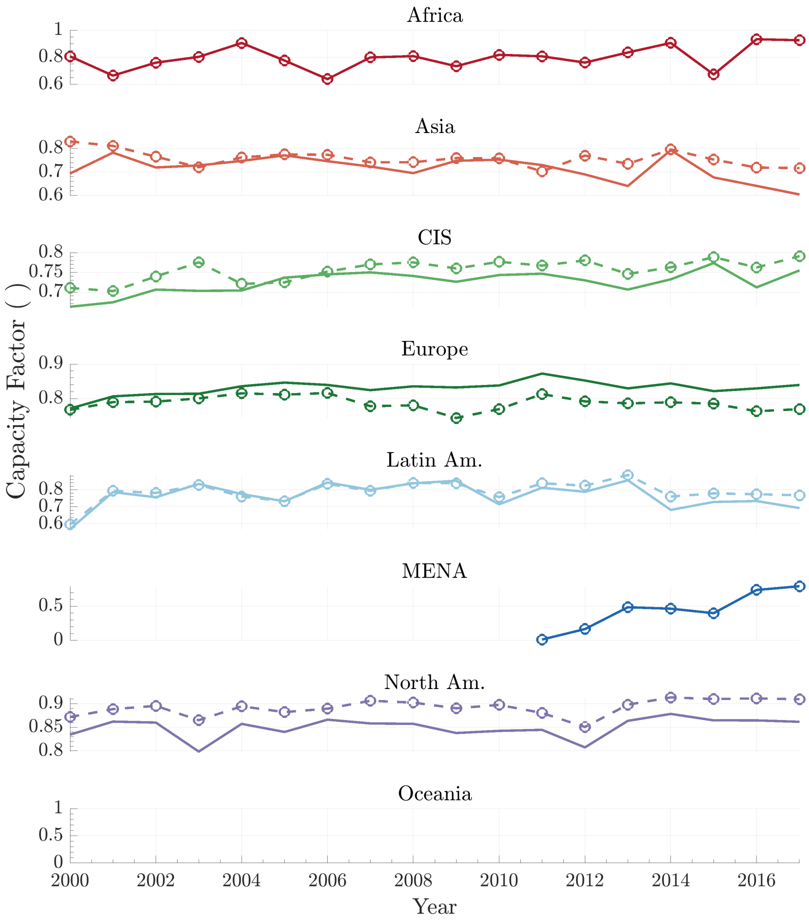

Fig. S22. Historical values for nuclear capacity factor.

F. Solar. Figure S23 displays the information used to calculate CF. The historical data showed in fig. S23a indicate the predominant solar electricity producing regions, Europe and Asia. In addition, fig. S23b shows the installed capacity that allows us to estimate the magnitude of electricity generated by solar PV. We observe that despite the fact that the United States pioneered solar power, Europe led until recently when it was surpassed by Asia.

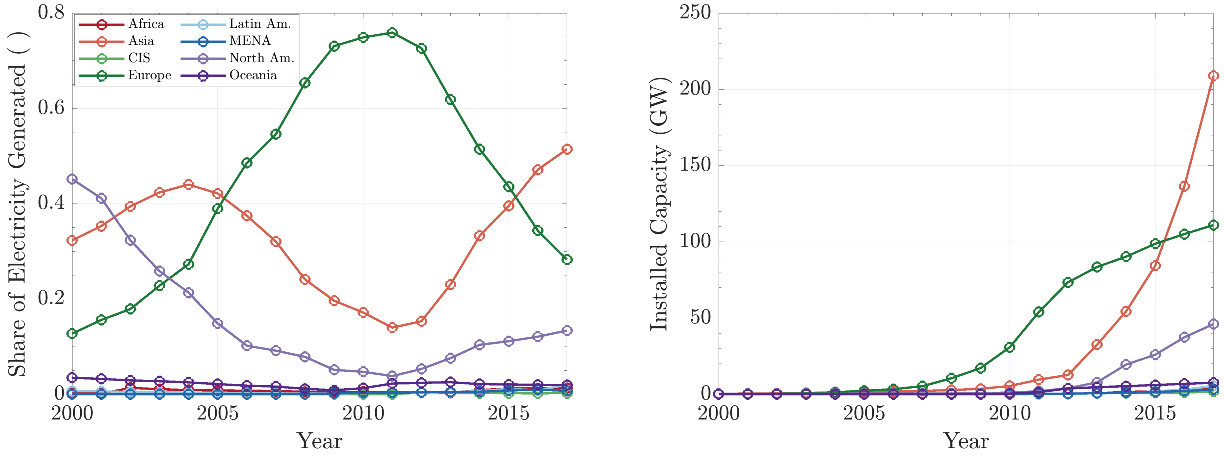

(a) (b)

Fig. S23. Solar. a) Electricity generation share from solar for the regions in fig. S6. b) Nominal installed capacity. Data from EIA(13).

Figure S24 shows the average CF and the weighted mean CF. It shows the distribution of CFs, and the weighted mean illustrates performance of big producers. Solar technology has few aspects that must be considered in the CF estimations. First, a broad expansion has occurred recently, implying that this technology will no longer be deployed at optimal locations, and the solar PV CF will decrease as a consequence. Some regions are more suitable and do not suffer from severe impacts due to seasonality, which explains the variance of reported values.

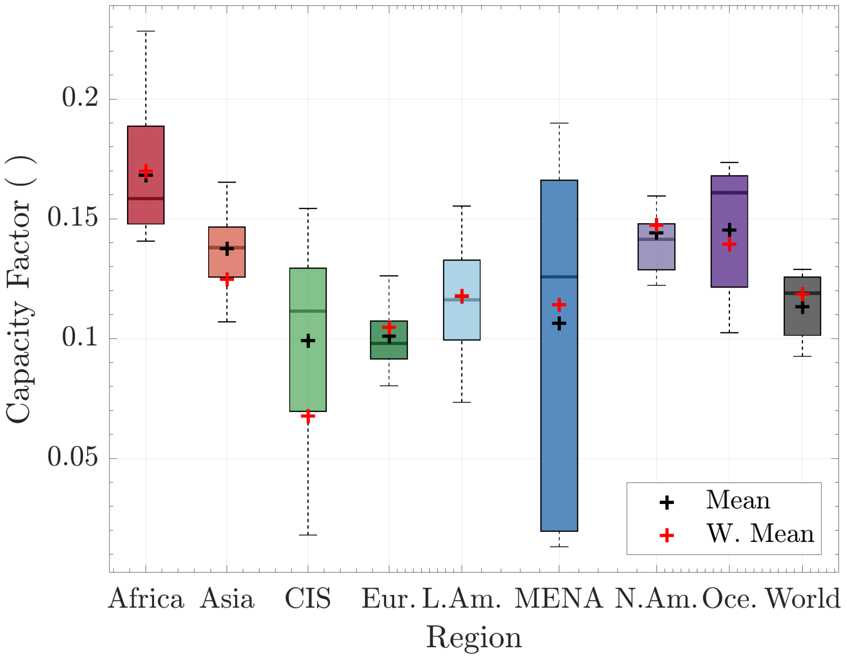

Fig. S24. Average values of solar capacity factor for the regions in fig. S6.

Figure S25 shows the historical values of the CFs and the weighted CFs. While fig. S24 shows the average values, fig. S25 presents the full data, allowing us to observe the dynamic evolution. For solar PV, we observe that mean and weighted mean

are close in the majority of the regions. In the case of Oceania, after 2010, more countries adopted solar power generation, but as previously mentioned, Australia is the heavyweight in this region. North America has a higher mean because of the U.S. Virgin Islands that reports capacity factors in the range of 0.25.

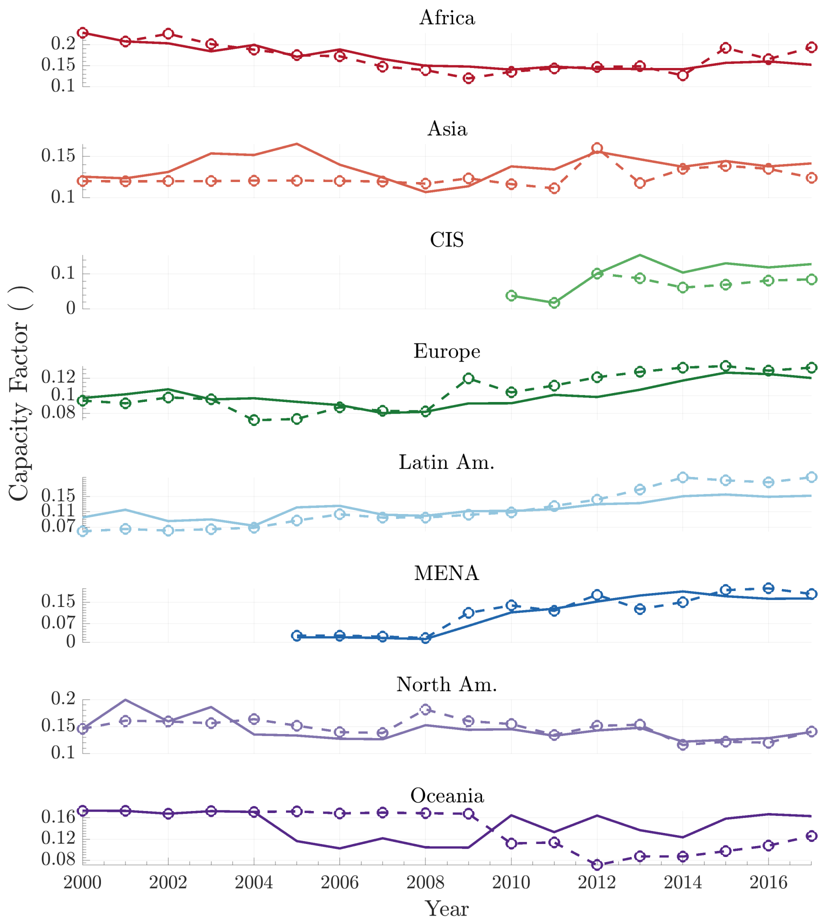

Fig. S25. Historical values for solar capacity factor.

G. Wind. Figure S26 display the information used to calculate CF. The historical data showed in fig. S26a indicate the key region that produces electricity from wind. In this case, wind electricity production started predominantly in Europe, followed by North America, and recently Asia is in the lead. In addition, fig. S26b shows the installed capacity that allows us to estimate the magnitude of electricity generated by wind turbines. We observe that Europe was the leading region in installed capacity, and was recently surpassed by Asia.

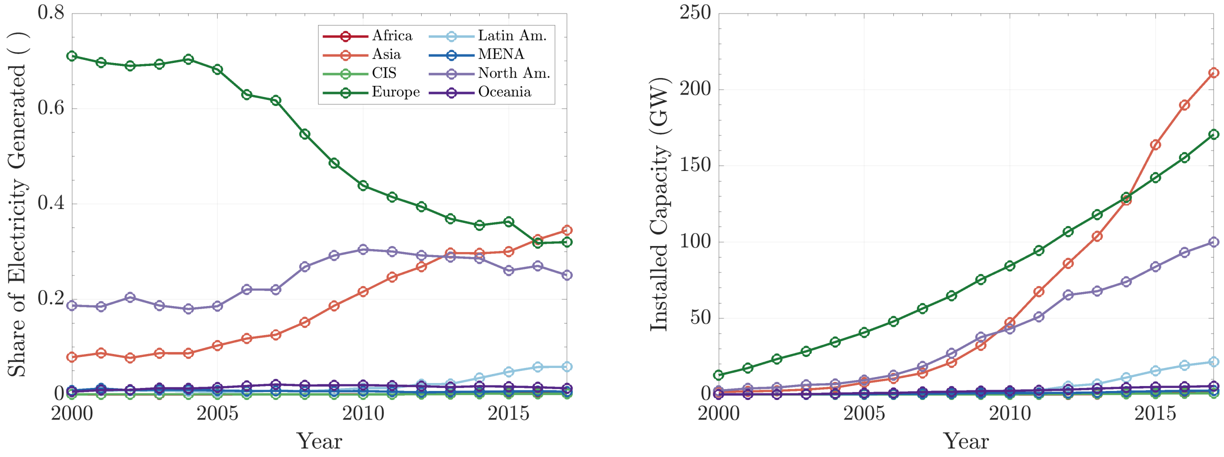

(a) (b)

Fig. S26. Wind. a) Electricity generation share from biomass for the regions in fig. S6. b) Nominal installed capacity. Data from EIA(13).

Figure S27 shows the average CF and the weighted mean CF. It shows the distribution of capacity factors, and the weighted mean illustrates the performance of big producers. An analysis of the information shown in fig. S26 shows that North America has notably less installed capacity, but and the share of electricity generated by wind is similar, which is caused by the higher CF in comparison to Asia and Europe. It is a good illustration of how different technologies are suitable for each region.

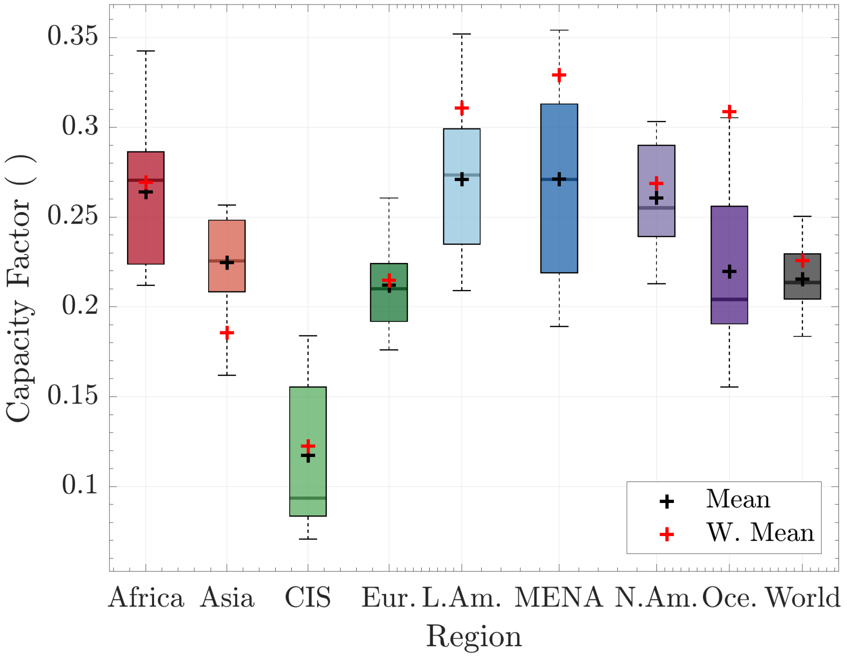

Fig. S27. Average values of wind capacity factor for the regions in fig. S6.

Figure S28 shows the historical values of the CF and weighted CF. While fig. S27 shows the average values, fig. S28 presents the full data, allowing us to observe the dynamic evolution. Interestingly, in the majority of regions, the mean and weighted mean close. In Oceania, the weighted mean is higher than the mean, which is opposite to Asia.

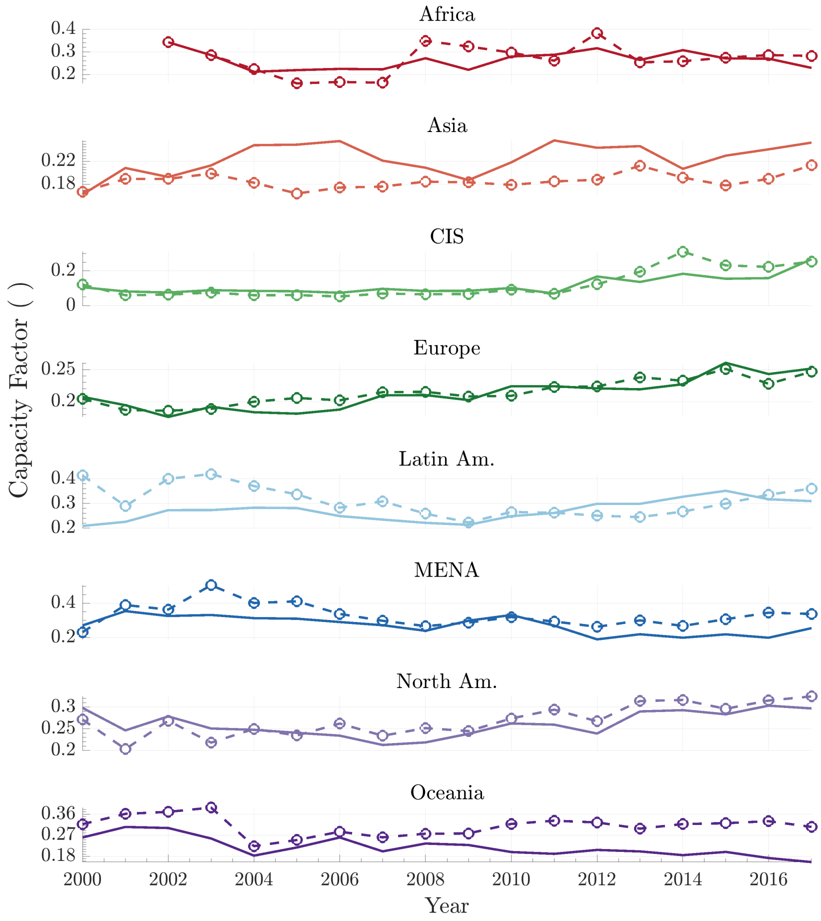

Fig. S28. Historical values for wind capacity factor.

8. Capacity Factor by Country

Table S1 presents each country's CFs for the main sources of electricity. Data treatment eliminates CF values equal to or greater than one or equal to zero. Beyond this filtering, the suspicious values associated with the quality of reported data have been retained. Some examples are the CF of South Sudan for solar PV (0.71), and for wind, Dominica (0.57) and Madagascar (0.57). In addition, other countries have CFs that seem to be out of range, and proper judgment should be applied before using them. Figure S29 shows the data histograms for all sources. This figure should be consulted as a reference to identify outliers mainly for solar PV and wind. For example, it is physically impossible to have an annual capacity factor for solar PV higher than 0.42.

Table S1. Capacity factor by countries

Country	Biomass	Fossil	Geothermal	Hydro	Nuclear	Solar	Wind
Afghanistan	-	0.14	-	0.24	-	0.2	0.16
Albania	-	0.06	-	0.36	-	0.11	-
Algeria	-	0.46	-	0.10	-	0.10	0.16
American Samoa	-	0.42	-	-	-	-	-
Angola	0.25	0.29	-	0.46	-	0.16	-
Antarctica	-	-	-	-	-	-	0.19
Antigua and Barbuda	-	0.53	-	-	-	0.15	-
Argentina	0.34	0.39	-	0.44	0.72	0.19	0.25
Armenia	-	0.12	-	0.19	0.68	0.13	0.12
Aruba	0.27	0.46	-	-	-	0.16	0.47
Australia	0.43	0.53	-	0.28	-	0.14	0.28
Austria	0.22	0.39	0.11	0.54	-	0.09	0.20
Azerbaijan	0.48	0.41	-	0.24	-	0.14	0.09
Bahrain	-	0.78	-	-	-	0.18	0.16
Bangladesh	0.15	0.61	-	0.46	-	0.15	0.21
Barbados	-	0.48	-	-	-	0.17	-
Belarus	0.26	0.39	-	0.35	-	0.06	0.15
Belgium	0.56	0.42	-	0.34	0.82	0.09	0.21
Belize	0.31	0.10	-	0.43	-	0.14	-
Benin	-	0.13	-	0.01	-	0.09	-
Bermuda	-	0.44	-	-	-	-	-
Bhutan	-	0.01	-	0.54	-	0.17	0.11
Bolivia	0.21	0.35	-	0.54	-	0.13	0.29
Bosnia and Herz.	0.49	0.48	-	0.28	-	0.17	0.38
Botswana	-	0.55	-	-	-	0.10	-
Brazil	0.43	0.38	-	0.52	0.79	0.11	0.25
British Virgin Is.	-	0.40	-	-	-	0.14	0.11
Brunei	-	0.48	-	-	-	0.11	-
Bulgaria	0.37	0.50	-	0.19	0.77	0.12	0.17
Burkina Faso	0.23	0.27	-	0.36	-	0.17	-
Burma	0.29	0.36	-	0.44	-	0.17	-
Burundi	0.18	0.18	-	0.34	-	0.15	-
Cabo Verde	-	0.32	-	-	-	0.10	0.28
Cambodia	0.27	0.38	-	0.30	-	0.08	0.11
Cameroon	-	0.30	-	0.61	-	0.15	-
Canada	0.60	0.45	-	0.56	0.80	0.13	0.25
Cayman Islands	-	0.46	-	-	-	0.16	-
Central African Rep.	-	0.10	-	0.67	-	0.15	-
Chad	-	0.43	-	-	-	0.15	-
Chile	0.72	0.38	0.30	0.47	-	0.21	0.27
China	0.46	0.51	0.57	0.39	0.79	0.12	0.19
Colombia	0.66	0.33	-	0.51	-	0.08	0.34
Comoros	-	0.34	-	0.40	-	-	-
Congo-Brazzaville	-	0.19	-	0.48	-	0.09	-
Congo-Kinshasa	-	0.07	-	0.35	-	0.16	-
Cook Islands	-	0.38	-	-	-	0.10	-
Costa Rica	0.35	0.08	0.77	0.50	-	0.02	0.40
Croatia	0.42	0.33	-	0.39	-	0.11	0.20

Table S1 continued from previous page

Country	Biomass	Fossil	Geothermal	Hydro	Nuclear	Solar	Wind
Cuba	0.12	0.35	-	0.20	-	0.10	0.18
Cyprus	0.49	0.38	-	-	-	0.15	0.13
Czech Republic	0.62	0.48	-	0.23	0.76	0.09	0.16
Côte d'Ivoire	-	0.67	-	0.32	-	0.10	-
Denmark	0.49	0.29	-	0.26	-	0.09	0.25
Djibouti	-	0.27	-	-	-	0.19	-
Dominica	-	0.39	-	0.46	-	0.11	0.57
Dominican Republic	0.34	0.51	-	0.32	-	0.15	0.29
Ecuador	0.31	0.34	-	0.51	-	0.14	0.26
Egypt	0.54	0.55	-	0.54	-	0.03	0.34
El Salvador	0.31	0.34	0.77	0.40	-	0.07	-
Equatorial Guinea	-	0.70	-	0.37	-	-	-
Eritrea	-	0.27	-	-	-	0.15	0.23
Estonia	0.39	0.46	-	0.50	-	0.10	0.22
Eswatini	0.28	0.33	-	0.42	-	0.14	-
Ethiopia	0.20	0.05	0.10	0.45	-	0.19	0.27
Falkland Islands	-	0.16	-	-	-	-	0.43
Faroe Islands	-	0.26	-	0.32	-	-	0.37
Fiji	0.21	0.30	-	0.48	-	0.13	0.09
Finland	0.70	0.33	-	0.50	0.93	0.08	0.21
France	0.58	0.22	0.57	0.37	0.74	0.10	0.21
French Guiana	0.58	0.21	-	0.58	-	0.13	-
French Polynesia	-	0.32	-	0.44	-	0.13	-
Gabon	-	0.36	-	0.51	-	-	-
Gambia	-	0.41	-	-	-	0.17	0.01
Georgia	-	0.14	-	0.30	-	-	0.26
Germany	0.61	0.51	0.40	0.54	0.85	0.09	0.18
Ghana	0.26	0.38	-	0.55	-	0.08	-
Gibraltar	-	0.46	-	-	-	-	-
Greece	0.44	0.54	-	0.21	-	0.11	0.25
Greenland	-	0.16	-	0.58	-	-	-
Grenada	-	0.48	-	-	-	0.08	-
Guadeloupe	0.24	0.39	0.74	-	-	0.14	0.26
Guam	-	0.36	-	-	-	0.17	-
Guatemala	0.45	0.28	0.63	0.48	-	0.12	0.30
Guinea	-	0.20	-	0.41	-	0.16	-
Guinea-Bissau	-	0.14	-	-	-	-	-
Guyana	0.26	0.31	-	-	-	0.12	-
Haiti	-	0.26	-	0.35	-	0.10	-
Honduras	0.25	0.47	0.30	0.49	-	0.12	0.35
Hong Kong	-	0.33	-	-	-	-	0.13
Hungary	0.47	0.31	0.04	0.45	0.86	0.09	0.19
Iceland	0.29	0.00	0.83	0.71	-	-	0.33
India	0.45	0.56	-	0.37	0.64	0.18	0.17
Indonesia	0.02	0.50	0.86	0.30	-	0.02	0.46
Iran	0.27	0.44	-	0.20	0.44	0.04	0.25
Iraq	-	0.43	-	0.16	-	0.18	-
Ireland	0.76	0.45	-	0.35	-	0.12	0.26
Israel	0.51	0.47	-	0.40	-	0.15	0.20
Italy	0.56	0.37	0.88	0.35	-	0.10	0.18
Jamaica	0.41	0.53	-	0.62	-	0.07	0.28
Japan	0.81	0.44	0.61	0.42	0.53	0.12	0.19
Jordan	0.30	0.51	-	0.50	-	0.20	0.29
Kazakhstan	0.38	0.48	-	0.39	-	0.12	0.15
Kenya	0.34	0.39	0.82	0.48	-	0.15	0.36
Kiribati	-	0.50	-	-	-	0.12	-
Kosovo	-	0.40	-	0.31	-	0.01	0.30
Kuwait	-	0.43	-	-	-	0.10	0.11

Table S1 continued from previous page

Country	Biomass	Fossil	Geothermal	Hydro	Nuclear	Solar	Wind
Kyrgyzstan	-	0.22	-	0.47	-	-	-
Laos	0.01	0.37	-	0.51	-	0.15	-
Latvia	0.50	0.28	-	0.21	-	0.09	0.21
Lebanon	-	0.68	-	0.29	-	-	0.21
Lesotho	-	-	-	0.69	-	-	-
Liberia	-	0.31	-	-	-	0.17	-
Libya	-	0.43	-	-	-	0.18	-
Lithuania	0.41	0.11	-	0.42	0.71	0.09	0.22
Luxembourg	0.66	0.53	-	0.34	-	0.08	0.17
Macau	-	0.30	-	-	-	-	-
Madagascar	0.57	0.24	-	0.62	-	0.20	0.57
Malawi	0.43	0.98	-	0.57	-	-	-
Malaysia	0.11	0.51	-	0.34	-	0.14	-
Maldives	-	0.34	-	-	-	0.06	0.23
Mali	0.17	0.27	-	0.43	-	0.13	-
Malta	0.24	0.37	-	-	-	0.12	0.46
Martinique	0.62	0.39	-	-	-	-	0.19
Mauritania	-	0.38	-	0.34	-	0.16	0.36
Mauritius	0.69	0.31	-	0.18	-	0.14	0.29
Mexico	0.39	0.54	0.78	0.31	0.79	0.10	0.21
Moldova	0.55	0.69	-	0.62	-	0.11	0.21
Mongolia	-	0.45	-	0.23	-	0.13	0.22
Montenegro	-	0.63	-	0.30	-	-	0.15
Montserrat	-	0.50	-	-	-	-	-
Morocco	0.62	0.50	-	0.13	-	-	0.33
Mozambique	0.39	0.12	-	0.75	-	0.01	-
Namibia	-	0.03	-	0.61	-	0.17	0.47
Nauru	-	0.48	-	-	-	-	-
Nepal	-	0.03	-	0.53	-	0.17	-
Netherlands	0.64	0.44	-	0.30	0.90	0.08	0.22
Netherlands Antilles	-	0.50	-	-	-	-	0.43
New Caledonia	-	0.50	-	0.51	-	-	0.14
New Zealand	0.57	0.48	0.87	0.51	-	0.12	0.36
Nicaragua	0.30	0.39	0.40	0.37	-	0.15	0.40
Niger	-	0.22	-	-	-	0.14	-
Nigeria	0.29	0.32	-	0.35	-	0.17	0.11
Niue	-	0.29	-	-	-	-	-
North Korea	-	0.18	-	0.30	-	0.15	0.19
North Macedonia	0.75	0.51	-	0.28	-	0.12	0.32
Norway	0.38	0.29	-	0.52	-	0.18	0.25
Oman	-	0.45	-	-	-	-	-
Pakistan	0.65	0.46	-	0.49	0.61	0.17	0.30
Palestine	0.29	0.32	-	-	-	-	-
Panama	0.24	0.36	-	0.48	-	0.07	0.18
Papua New Guinea	0.14	0.54	0.88	0.47	-	0.18	-
Paraguay	0.40	0.09	-	0.75	-	-	-
Peru	0.61	0.26	-	0.67	-	0.13	0.17
Philippines	0.22	0.43	0.62	0.31	-	0.12	0.22
Poland	0.86	0.53	-	0.45	-	0.08	0.22
Portugal	0.63	0.43	0.75	0.29	-	0.16	0.24
Puerto Rico	0.21	0.44	-	0.13	-	0.07	0.16
Qatar	0.33	0.50	-	-	-	0.17	-
Reunion	0.75	0.46	-	0.48	-	0.13	0.11
Romania	0.48	0.28	-	0.29	0.90	0.11	0.26
Russia	0.25	0.45	0.60	0.41	0.77	0.11	0.09
Rwanda	0.17	0.35	-	0.30	-	0.11	-
Saint Helena	-	0.16	-	-	-	-	-
Saint Kitts and Nevis	-	0.36	-	-	-	-	0.42

Table S1 continued from previous page

Country	Biomass	Fossil	Geothermal	Hydro	Nuclear	Solar	Wind
Saint Lucia	-	0.50	-	-	-	0.11	-
Saint Pierre and Miq.	-	0.19	-	-	-	-	0.23
Saint Vincent/Gren.	-	0.33	-	0.40	-	0.10	-
Samoa	-	0.30	-	0.43	-	-	0.02
Sao Tome and Prin.	-	0.39	-	0.24	-	-	-
Saudi Arabia	-	0.50	-	-	-	0.20	0.19
Senegal	0.49	0.45	-	0.45	-	0.17	-
Serbia	0.52	0.64	-	0.50	-	0.11	0.15
Seychelles	-	0.37	-	-	-	0.11	0.13
Sierra Leone	0.01	0.25	-	0.34	-	0.17	-
Singapore	0.80	0.45	-	-	-	0.16	-
Slovakia	0.49	0.29	-	0.30	0.84	0.11	0.19
Slovenia	0.56	0.47	-	0.44	0.90	0.13	0.13
Solomon Islands	0.33	0.29	-	-	-	0.11	-
Somalia	-	0.47	-	-	-	0.09	0.09
South Africa	0.16	0.62	-	0.24	0.80	0.15	0.32
South Korea	0.44	0.58	-	0.25	0.86	0.13	0.19
South Sudan	-	0.70	-	-	-	-	-
Spain	0.62	0.38	-	0.25	0.87	0.16	0.23
Sri Lanka	0.22	0.46	-	0.33	-	0.13	0.19
Sudan	0.15	0.36	-	0.47	-	0.14	-
Suriname	0.34	0.36	-	0.57	-	0.15	-
Sweden	0.32	0.11	-	0.47	0.76	0.08	0.24
Switzerland	0.72	0.20	-	0.38	0.87	0.08	0.14
Syria	0.54	0.44	-	0.26	-	-	-
Taiwan	0.91	0.58	-	0.25	0.85	0.11	0.27
Tajikistan	-	0.08	-	0.41	-	-	-
Tanzania	0.04	0.46	-	0.45	-	0.19	-
Thailand	0.24	0.54	0.61	0.23	-	0.10	0.23
The Bahamas	-	0.42	-	-	-	0.14	-
Togo	-	0.17	-	0.32	-	0.15	-
Tonga	-	0.41	-	-	-	-	-
Trinidad and Tobago	0.49	0.50	-	-	-	0.16	-
Tunisia	-	0.44	-	0.13	-	0.10	0.29
Turkey	0.34	0.53	0.60	0.31	-	0.09	0.29
Turkmenistan	-	0.51	-	0.36	-	-	-
Turks and Caicos	-	0.41	-	-	-	-	-
U.S. Pacific Islands	-	0.96	-	-	-	-	-
U.S. Virgin Islands	-	0.30	-	-	-	0.11	-
Uganda	0.32	0.29	-	0.54	-	0.16	-
Ukraine	0.34	0.25	-	0.26	0.72	0.06	0.12
United Arab Emirates	0.55	0.45	-	-	-	0.14	0.21
United Kingdom	0.71	0.45	-	0.34	0.74	0.08	0.25
United States	0.64	0.43	0.72	0.39	0.90	0.15	0.27
Uruguay	0.37	0.18	-	0.53	-	0.13	0.22
Uzbekistan	-	0.44	-	0.43	-	0.14	-
Vanuatu	0.09	0.23	-	-	-	0.13	0.21
Venezuela	-	0.34	-	0.58	-	0.15	0.20
Vietnam	0.05	0.50	-	0.47	-	0.14	0.19
Western Sahara	-	0.17	-	-	-	-	-
Yemen	-	0.47	-	-	-	-	-
Zambia	0.27	0.11	-	0.61	-	0.17	-
Zimbabwe	0.15	0.30	-	0.72	-	0.12	-

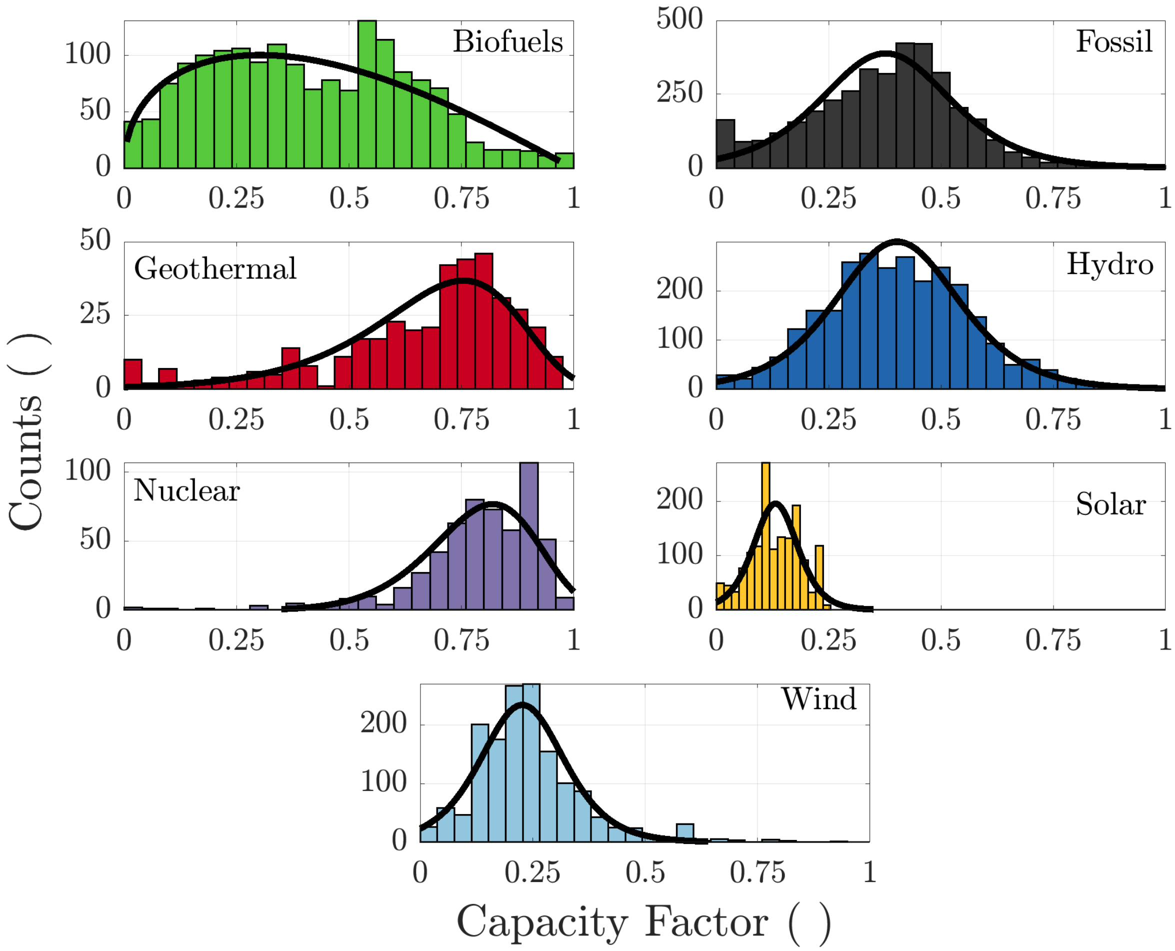

Fig. S29. Histograms of capacity factor. The binned data are for all the countries in table S1.

9. Solar

A. Detailed Performance. Here we provide the detailed performance over the analyzed periods for each of the three locations we picked: Austin, US; Càceres, ES; and Thuwal, SA. Table S2 shows the nominal capacity, the sun-tracking and the cleaning frequency. The installation in Austin is a domestic array; Càceres is a utility scale commercial array; and Thuwal is a utility scale array non-commercial.

Table S2. Details of solar arrays

Location	Nominal Capacity (kW_p)	Sun Track	Cleaning
Austin-US	16.36*	fixed - optimum angle	2×/year
Càceres-ES	950	Two-axis	2×/year
Thuwal-SA	1000	fixed - horizontal	Biweekly

* Composed of 3 ground arrays. Arrays 1 and 2 consist of 16 panels of 260W each, installed back in July 2013. Array 3 consists of 24 panels of 335 W, installed in July 2019 by the same company.

A.1. Austin-US. Figure S30 shows the detailed CF and fig. S30b the energy yield. Arrays 1 and 2 have the same specifications, and were installed back in 2013; array 3 was installed in 2019 with more modern PV panels, individual panel controllers and a full-wave inverter. We choose to show performance of these three arrays to demonstrate that: (1) arrays with the same specifications and in the same location can perform differently, because of somewhat different shading by the surrounding trees, and (2) technology has been improving. The differences among the array performances affect the calculated mean and standard deviation for their assembly. This kind of heterogeneity of systems must be expected and can affect significantly global estimates.

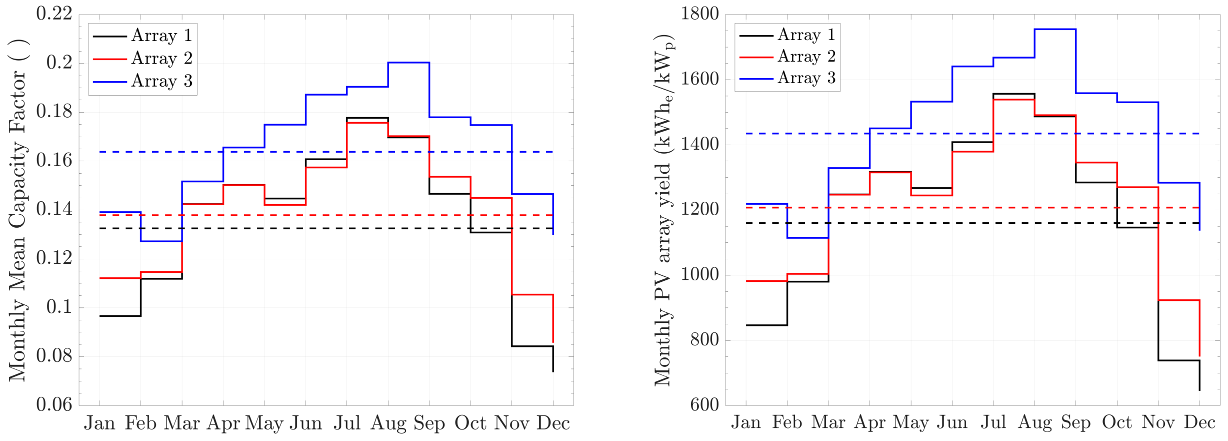

(a) (b)

Fig. S30. Detailed performance of the Austin, TX arrays. (a) Monthly capacity factor. (b) Energy yield, values are plotted as monthly stairs assuming same performance over each calendar year. For Arrays 1 and 2 we display the monthly means from 2014 to 2021. For Array 3 we show the monthly means for the years 2020 and 2021. The broken lines are the annual means.

A.2. Càceres-ES. We now show the detailed CF (fig. S31a) and energy yield (fig. S31b) in Càceres, Spain. This array is operated by Siemens for the second author. Its performance decreased by 10 % in 2020, compared with the other years. This efficiency decline was caused mostly by an irradiance decrease in 2020. Its sun-tracking system needs to be properly maintained to ensure a good performance. In this case, even a single six-month delay in cleaning the panels and tuning their tracking systems impact the array's performance.

We should add, that the Càceres location in Spain is relatively dust-free and very sunny. The only serious dust comes from the Sahara desert and from droughts.

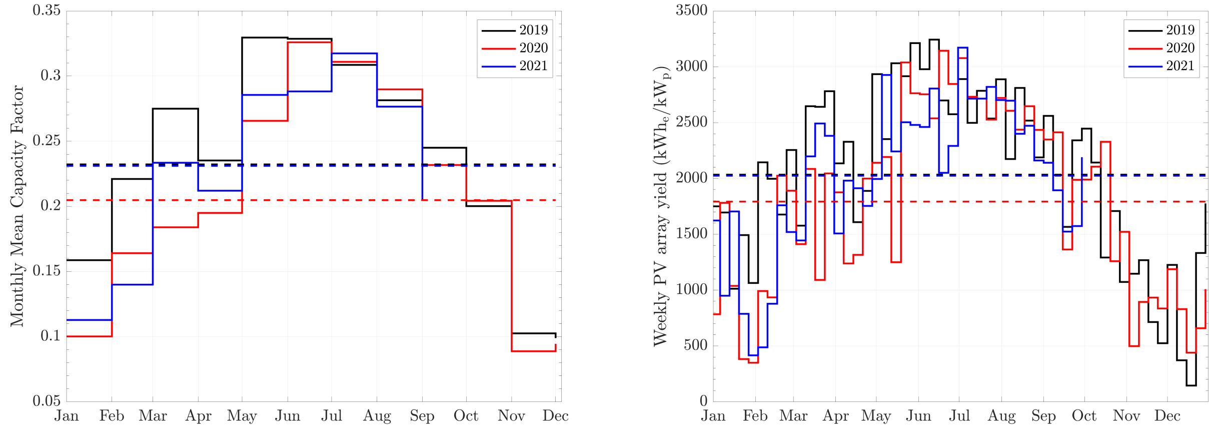

(a)

(b)

Fig. S31. Càceres detailed performance. (a) Monthly CF. (b) Energy yield is plotted as monthly stairs assuming a full year performance at that level.

A.3. Thuwal-SA. Finally, we show the detailed CF (fig. S32a) and energy yield (fig. S32b) of the KAUST roof-top array in Thuwal, SA. This array's performance between May and June 2020, (the red line) declined because of the COVID-19 lockdown. Severe dust deposition reduces drastically the solar array's performance. This array was installed in 2018 on top of a large research building with a flat roof. The panels are fixed and tilted 10 degree NS.

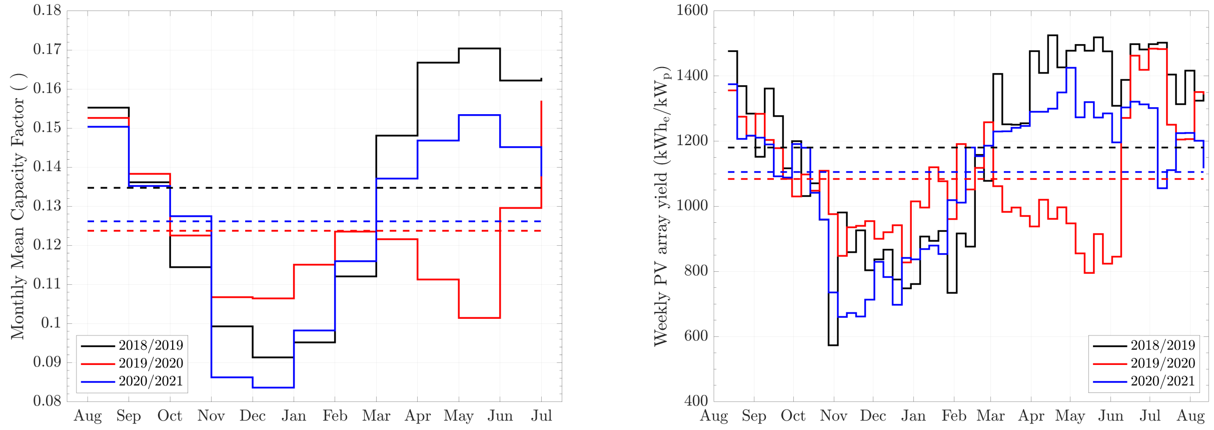

(a)

(b)

Fig. S32. Thuwal detailed performance. (a) Monthly capacity factor. (b) Energy yield is plotted as monthly stairs assuming a full year performance at that level.

Overall,these detailed data show that solar PV arrays are very sensitive to many environmental factors. Proper maintenance is essential to keeping these arrays performing well. In one case, we could clearly see the drop in performance that occurred in 2020, due to lack of maintenance imposed by COVID-19. The ground arrays in Austin were washed twice in 2020 by the senior author, who spent the COVID lockdown on his property.

B. Comparison of Performance. Figure S33 shows how the panels have been performing based on the local average incident irradiation. The average irradiation data was estimated using PVGIS(28) for the period from 2005 to 2020. The reference surface was optimally tilted in Austin; we used normal incident irradiation in Càceres, and horizontal surface in Thuwal – matching the array configurations.

Figure S33a shows the actual performance in Austin. The most recent array (Array 3) performs better than arrays 1 and 2. Overall, the arrays are delivering 64 % of the expected power output if we only consider incident radiation. Figure S33b shows the actual performance for the array in Càceres. The initial observation is that in some months, the performance was above the expected; in March 2019, by up to 20 %. These deviations are expected as March 2019 seems to have been an atypical month. Overall, the array in Càceres delivers 82 % of the expected performance, the highest yield of the three analyzed systems. Figure S33c shows the performance of the Thuwal array. In the best month, the array only delivered 58 % of the expected

output, and on average, it delivered 48 %. The Thuwal array, placed in the sunniest location has had the worst performance observed among the five compared arrays.

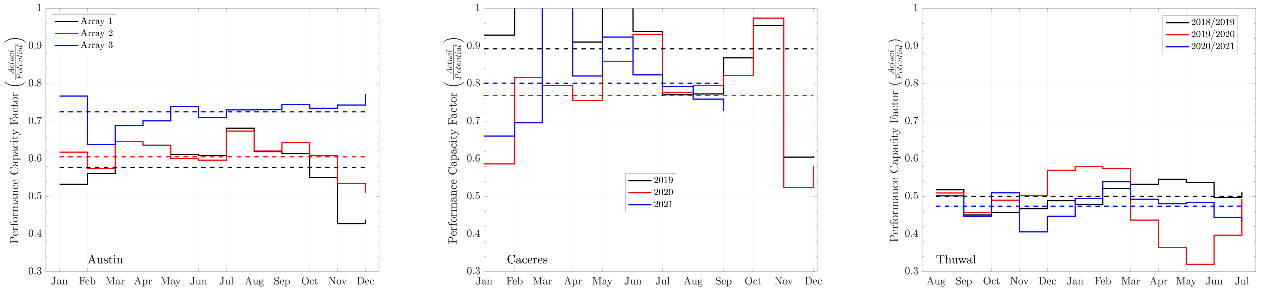

(a) **(b)** **(c)**

Fig. S33. Comparison of actual performance divided by potential performance for (a) Austin; (b) Càceres; and (c) Thuwal. The potential performance was estimated from average irradiance for each location over the period from 2005 to 2015. Irradiance data are from(28).

10. Economics of variable output renewables

Our main points about economics can be summarized as follows:

1. Paraphrasing somewhat Sir Eddington(29), when laws of Physics collide with laws of Economics the defeat of economics will be complete, and there is no hope of another outcome.

2. There is a critical capacity factor below which energy return on investment becomes too small, say 3-5:1, to run current complex infrastructure and physics destroys the system. Our case studies below show how this may happen.

3. The current 19 TW global primary power machine cannot run continuously on renewables alone. But with a sufficient decrease of use of primary power and electricity in developed countries, a significant simplification of the global economy, and *giant electrical power storage/backup systems*, renewables will continue to play an increasingly important role.

As to the first bullet, for every "engine" (power supply system) ever constructed there are numerous factors that contribute to its efficiency and hosts of reasons to choose one engine or another for particular purposes. Nevertheless, the second law of thermodynamics is of fundamental importance, because it places an unbreakable upper bound on efficiency, independent of all the complex factors one might like to invoke in a particular narrative.

While capacity factor is not quite as fundamental as the law of increase of entropy, see(29), its significance in setting limits is of comparable importance and for similar reasons. The practical limits we can now quantify, set by factors such as average wind speeds, cloud cover and dust, place specific limits on attempts to supplant the current energy mix in ways that no clever economic incentives or technological progress can possibly circumvent.

We are engineers, jointly with 26 years of experience in designing, installing, monitoring, maintaining and using solar PV panels. Thus, our own economic analysis is simplified, case-specific and applied to solar PV systems, but we will cite a few definitive papers that analyze economics of green transitions.

Let us start from Patzek's all-electric household as an example. Nominally, this household is fully sustainable, with 3 ground PV arrays that develop 16 kW_p, such that his cumulative electrical bill (credit) was -$1,127.00 on 05/30/2022. The Patzeks use only the rainwater collected from the metal roofs of four structures into 4 tanks with the total capacity of 250 m^3, have an 8 kW_p passive solar heater for all hot water needs, and have a high-efficiency, 23 kW_p, wood stove capable of heating the entire house in winter with wood gathered from the trees on their 15 acre property. The Patzeks also have two water wells, but no city water connection and no sewage service, and only the non-recyclable waste leaves their property, so little of it that they share trash pickup with a neighbor. Multiple sensors allow them to track remotely how their household functions. While on the average the Patzeks export roughly 50% of the electricity they produce, they *really need* external grid electricity at least 12h a day, and more during winter, see Figure S34.

Now, let's go into the simplified economics of Patzek's top-of-the-line, monocrystalline PV arrays, made in Taiwan with real 25-year warranties. The first two arrays were installed in 2013, at the total cost of $27,603, including the eGauge sensors, or \$3.50/W_p, \$2.62/W_p after the federal tax credit. Their powerful galvanized steel frames were cemented 6 ft deep in the drillholes in limestone rock, and about 200 ft of cable trenches had to be excavated out. Patzek also paid an additional $6000 to build a solar house for the inverters and future large battery packs. The Patzek arrays paid for their embedded energy in roughly 6 years, based on the assumptions in(32) applied to them. The third, superior array was installed in 2019, at a cost of $29,370 or \$3.62/W_p, \$2.56/W_p after the federal tax credit. It paid back its embedded energy in about 3 years. In both cases, frame materials, site preparation, hole drilling, cement trucks, labor, transport and electronics were the major costs, in addition to the modules. The average electricity cost/income has been $0.1033/kWh (PEC in Texas) for the Patzeks. At this electricity cost, their arrays that have been producing 22 MWh/year on the average, would pay the initial investment back in

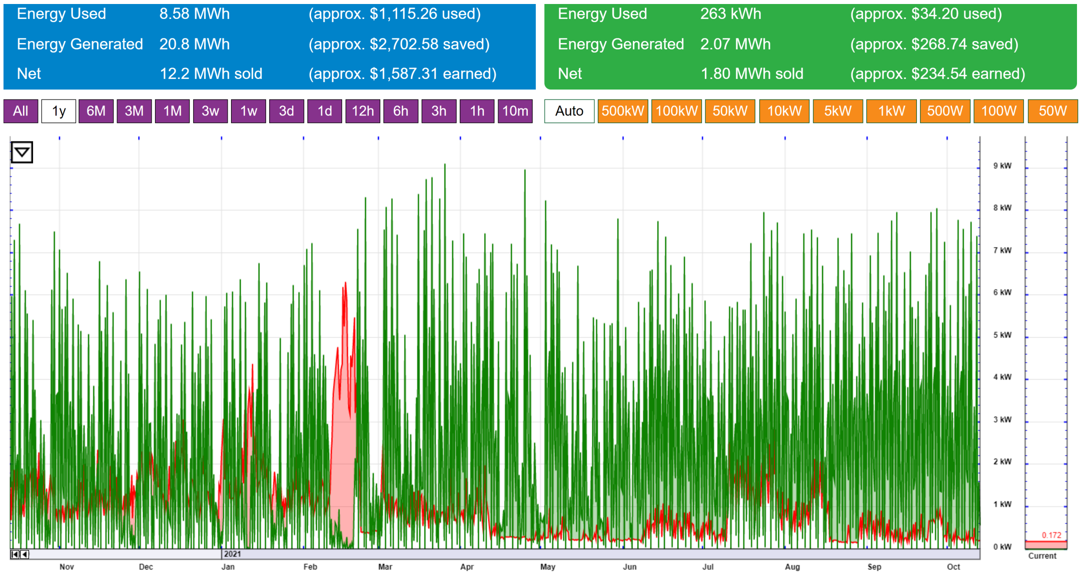

Fig. S34. Twelve months of functioning of Patzek's PV arrays (green) and electricity use (red) from the eGauge sensors. Because of averaging, the peak power appears to be much lower that the 13 kW these arrays produce at noon. The most valuable electricity is the one missing in emergency. Please notice the deep-freeze in Austin in February 2021. During that most severe weather, the coal-powered electrical grid never failed at the Patzeks' house, while their solar arrays delivered almost nothing for 9 days, for the reasons shown in Figure S35. The February 2021 freeze in Texas(30) and the summer 2022 panic in Europe(31) have common elements of fear of the cold winter snaps, and possibly insufficient natural gas, coal and fission to power the grid 24/7.

,

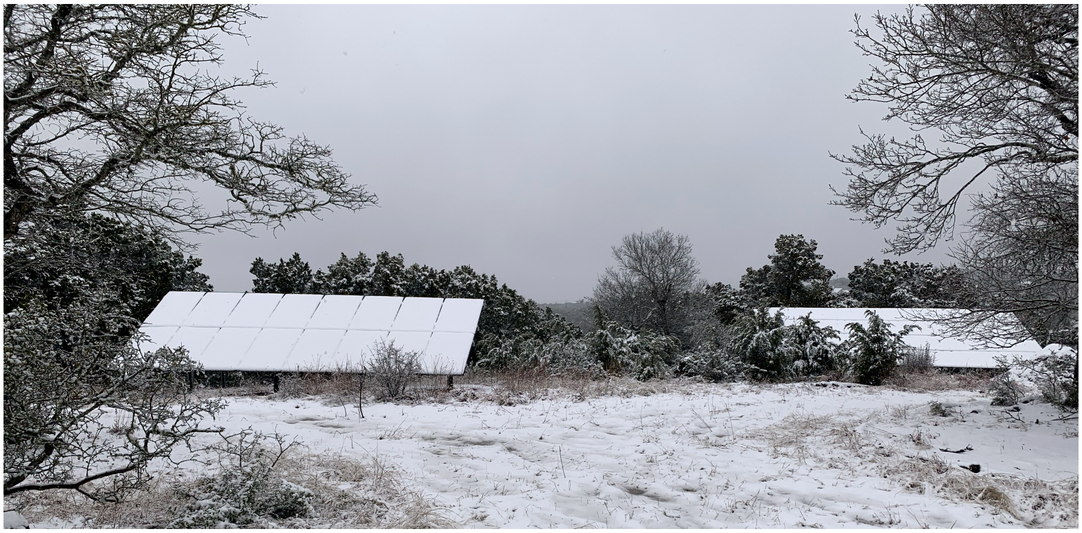

Fig. S35. For nine days, Patzek's arrays were completely covered with a thick layer of ice and snow on top. The Patzeks lost much of their rainwater infrastructure, including two pumps that froze, simply because the 1 kW electric heaters they had in each pumphouse were set to turn off a 3°C, too low to counteract the prolonged -15°C temperature. This unprecedented polar air blast was a direct result of climate change.

$((27603 + 29370)(1 - 0.26) + 6000)/22000/0.1033 = 21$ years, or never in practice. But economic payout was *never* the Patzeks' goal. Changing lifestyles and living more renewable lives were. And here the Patzeks have succeeded beyond their wildest expectations.

As we have just shown, focusing on the PV module prices alone can be insufficient; other costs can more than offset the falling module prices. If PEC charged the Patzeks $0.33-0.49 kWh on average, as PG&E would in Northern California, or if they lived in Germany, the payback time would have been 4.5 – 6.6 years.

Our friend, Chris Vernon, is a key member of a sustainable agroecology community in Wales. Their 700 kW wind turbine is being paid ∼£100/MWh directly from the utility (Power Purchase Agreement, PPA). But they also receive a Feed-in-Tariff (FiT) payment of around £20/MWh. This payment is funded by a levy on energy bills. Additionally they receive a Renewable Energy Guarantees of Origin (REGO) payment of £1.80/MWh, and a Generator Distribution Use of System (GDUoS) payment

of £5/MWh (again levied on bills). In USD, they are being paid together 16.5 cents/kWh, which is quite low. However, the wholesale market has been very volatile. Up until a few months ago that £100/MWh was just £60/MWh, and Chris just heard of a local farmer with a similar turbine, who signed a PPA at £247.80/MWh. On August 18, 2022, Chris communicated this: “That [price], £100 /MWh, I mentioned last month [is obsolete] – we're currently being offered ∼£350 /MWh!”

Chris's community is in the process of adding 200 kW of solar PV at the foot of the turbine to make use of the grid connection cable and the fact that it's rarely very sunny and windy at the same time. For that they will get the same PPA price, REGO and GDUoS payment, but not the FiT payment.

In closing, if the Patzeks were to opt for an off-grid battery solution that would power some of their necessary air conditioning for up to 12 hrs/day, their additional cost would have been $40-60K, depending on the batteries, controllers and installation costs. These batteries might have failed during the 9-day deep freeze of 2021. Thus, the fragile, expensive battery backup systems cannot replace base load electricity from the grid, but with significant changes in lifestyle they might suffice in good enough weather. The PV systems the Patzeks have are inaccessible without ample land and large upfront investment. These requirements make it impossible for most people in the US to install comparable arrays.

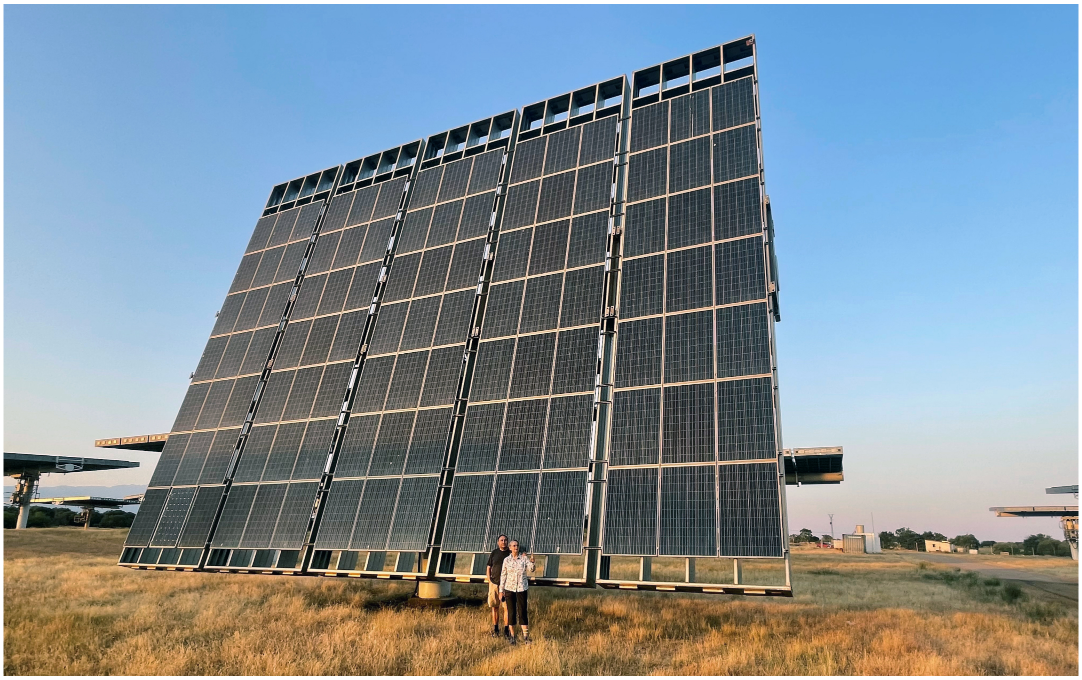

Fig. S36. The Patzeks in front of one of the 38 PV arrays on 6 ha of Pedro Prieto's Càceres property. The original 2007 design, at 6 €/W$_p$, delivered the bi-axial tracking high-concentration PV (HCPV or CPV) arrays by Amonix. Their PPMA Fresnel lenses deteriorated in sunlight and heat, and the bi-axial hydraulic systems were incapable of maintaining precision required for the Fresnel lenses to focus the sunlight on the modules. In 2014, at an additional cost of 1 €/W$_p$, the Amonix panels were replaced with polycrystalline ones rated at 950 kW$_p$. The array in the photo is not parked in the rest position, because its hydraulic tracking system has failed.

A view of the commercial PV installation in Càceres, Spain, is shown in Figure S36. This particular project consists of 38 biaxial tracking arrays on 6 ha of Pedro Prieto's property. In 2007, the original design was a high-concentration (400 suns) PV system by Amonix with plastic Fresnel lenses. This system was later improved and industrialized by the Spanish Basque company Guascor Solar, later Guascor Foton. The original contract granted the retail price of electricity of the order of 0.28 €/kWh in today's euros for the first 25 years, and then a lower price for life. With these premium tariffs and record retail prices of electricity since 2007, the economic payback would have been seven years, even though the plant had been very expensive at 6 €/W$_p$, requiring a six million euro investment.

As the installed PV power increased exponentially in Spain, some 20 different decrees, regulations and laws were introduced, each limiting and voiding the previous ones, and lowering promised incomes. At the same time, the PV system prices were decreasing in parallel. So the equation in Spain was approximately that the economic payback could be around 7 to 10 years. Today, premium tariffs do not exist, but the utility-scale plants insist they can payback initial investment in about 10 years, and guarantee project life span of 30 years, which is unrealistic, because today there are almost no industrial or commercial companies offering a return of investment in 10 years.

Royal Decree (RD 477), pp. 47-48, column “Coste subvencionable unitario màximo (€/kW)” gives electricity price guarantees. This value is a reference for investment costs. Auctions Resolution is based on the RD 960/2020 and Order TED/1161/2020. In Annex 1, pages 5 and following, in the column “Precio de adjudicación (euros/MWh)” (Awarded Price in euros/MWh), the price on which the offers were awarded for the prices submitted (the lowest ones) are in the column “Tecnología” for solar PV. They are, approximately, between 25 and 35 €/MWh, or about 26-37 US cents/kWh.

Parenthetically, in(33), Chapter 5.3, page 42, IEA described an identical Amonix system. They mentioned a 25 kW system similar to that in Càceres, but with 500 suns concentration and 37% efficiency cells (Càceres was 400 suns and 27% efficient), but in no case, not one of the Amonix systems has performed as predicted so far, and only 100 MW_p were sold worldwide by academic theoreticians, who promised Energy Return on Investment (EROI) of 33 to 41:1, but never struggled with real life systems. By 2022, most if not all of these systems were abandoned or replaced. This is just one of the many difficulties with theoretical analyses that can be orthogonal to reality.

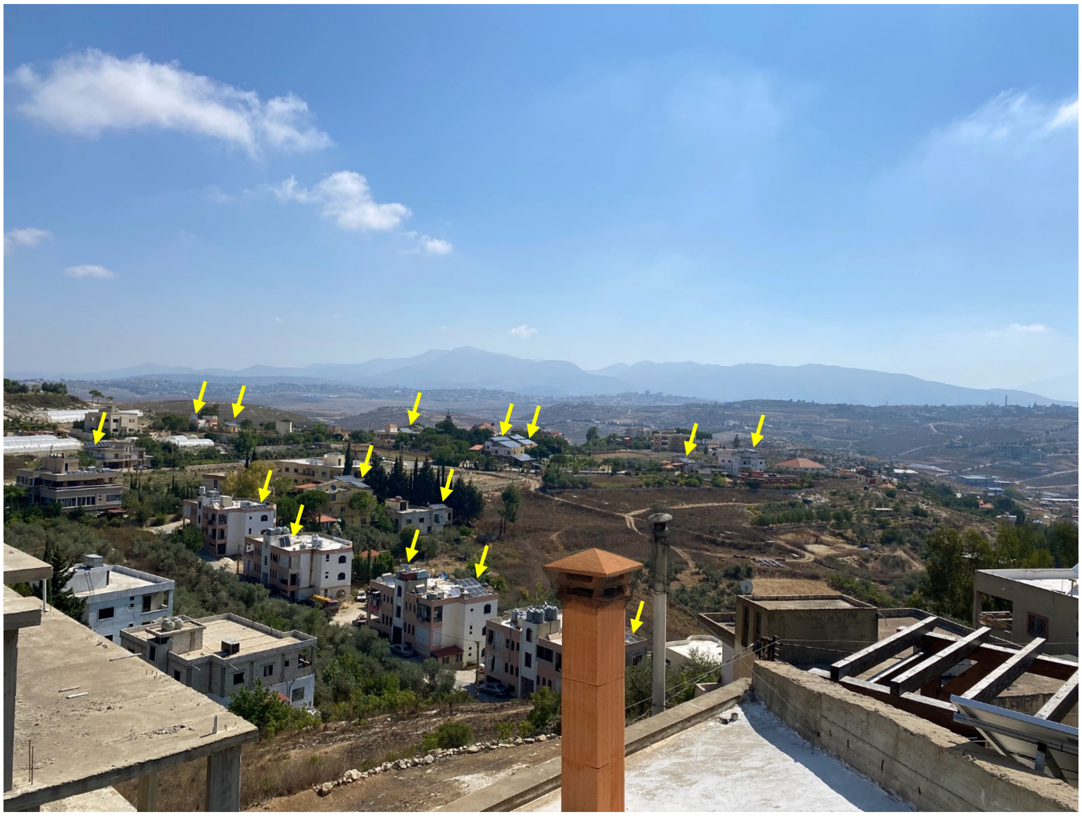

Fig. S37. A view of Doueir from the Hoteit family house roof. With no functioning grid, $> 80\%$ of the houses have already installed PV arrays highlighted with the arrows. This relatively affluent town may not be representative of the rest of Lebanon, but all these installations happened in the last 6 months and the process is moving fast. The most common systems that are installed now are 4 panels + a small inverter + 2×200 A lead-acid battery (mostly made/recycled in India with unknown reliability) at a cost of $3000. This is an illustration of how a real green revolution looks like up close in a relatively mild sunny climate.

The next example of an existential solar PV installation comes from our friend, Prof. Hussein Hoteit, who has parents in Lebanon. They live in Doueir (Figure S37), a small town near Nabatieh, about 70 km south of Beirut. The town has a Mediterranean climate, with an average annual temperature of 20 °C (as high as 32 °C in August). Their climate is much milder than those in Austin, Càceres and KAUST. Between 2009 and 2019, the local electric utility could provide only 6 hours of electricity per day, and it almost completely collapsed in 2022. The remainder of electricity supply was provided by a small private company that generated electricity for the neighborhood using an inefficient and expensive diesel generator. That company went bankrupt in early 2022, because of the collapse of the Lebanese pound (LBP) that was equivalent to 1/29,000 of USD on 7/12/2022, (a depreciation by a factor of 20 since 2019), increased fuel prices, and inability of people to pay. Consequently, out of absolute necessity, in April 2022, Hoteit installed a 14×600 $W_p = 8.4$ kW_p array of monocrystalline PV panels for his parents (Figure S38), together with a 10 kWh lithium ion battery (Figure S39). The total cost of this system was ~$10,000 ($4000 panels, $2000 battery, $2000 inverter, and $2000 wires + accessories + labor). This off-grid system powers all the household needs during the day (2 refrigerators, 2-3 AC units, stove, lights, and others up to 5KW). At night, the battery is enough to power two refrigerators, 2-3 LED lights and sometimes a fan, but nothing more.

Today in Lebanon, most people have no other options but to provide their own electricity. According to Hoteit, in August 2022, grid collapse in Lebanon was almost complete, with one hour of electricity per day on average. With his US-like salary, Hoteit was able to pay cash for his parents' system, but most people in Lebanon cannot. Lebanon is an example of a post-collapse state and society. In Lebanon, payback time and pricing are less relevant than the vital access to *any* electricity. What counts is the ability to have cash on hand for the initial steep investment, out of reach for most people. In Lebanon, Yemen, parts of Nigeria, Egypt, Somalia, Rwanda(34), Sri Lanka, Puerto Rico(35), and so many other developing/former colony countries(36), cheap home PV systems are the only means of accessing electricity that we in the developed countries took for granted in 2022, subject to rolling blackouts in cold winters and hot summers, like in Texas.

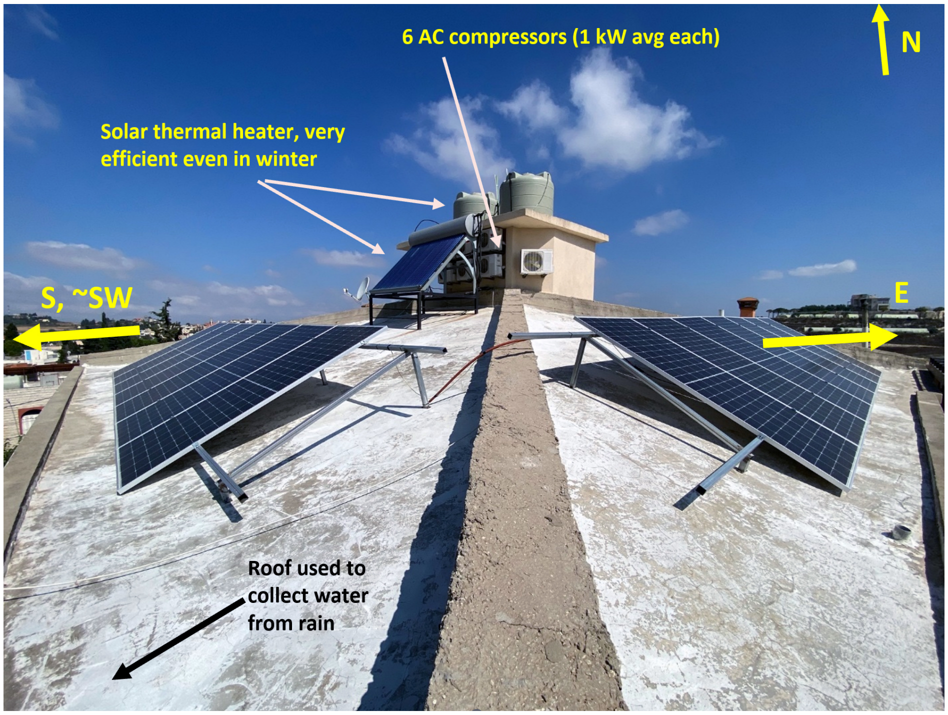

Fig. S38. Two PV arrays, each consisting of 7 double-size panels rated at 600 W_p/panel (8.4 kW_p total) are placed on the roof of Hoteit's house. One array faces east and the other one S-SW. The 6 kW_p solar heater satisfies all hot water needs of the family. Four of the six small 1 kW_{avg} AC units (2.6 kW to start, one per room) are visible. At any given time, only up to three units may be on. The concrete roof is used to collect rainwater into the underground concrete tanks ($25,000 to install), with the total storage capacity of 900 m^3. This rainwater is mostly used to irrigate the 1000 m^2 fruit and vegetable garden that feeds the family and is a source of income.

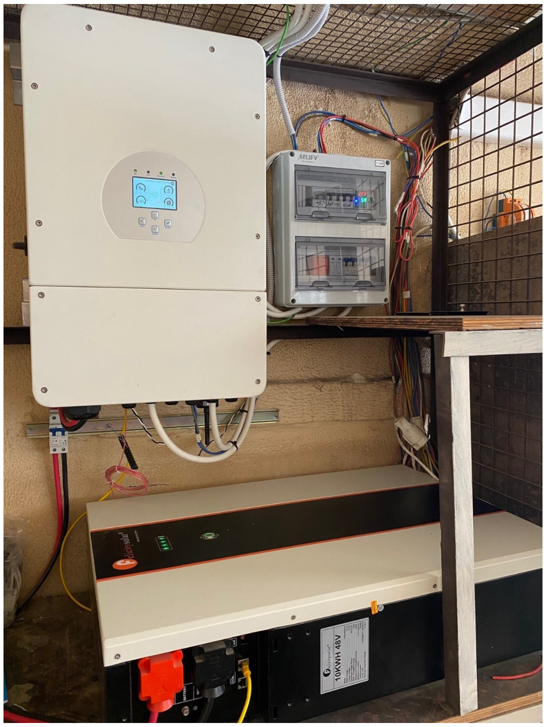

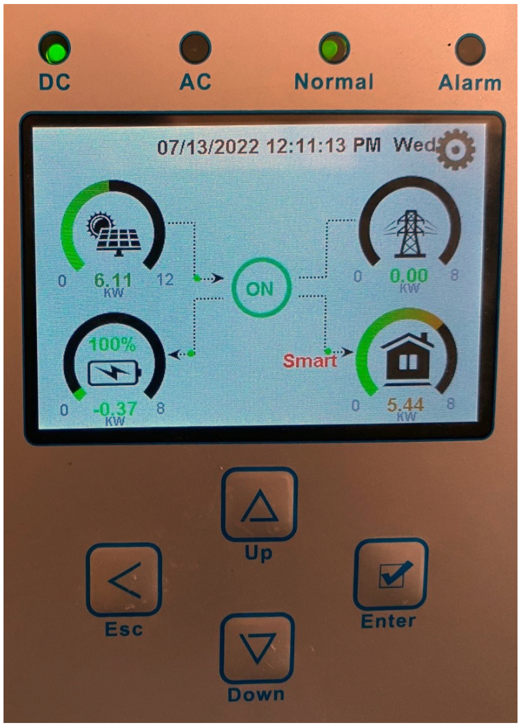

Fig. S39. (Left) The 10 kWh battery, inverter and controller. (Right) Power generation/consumption at 12:11 pm, when peak generation reached 6.11 kW (out of the nameplate capacity 8.4 kW). At this time, Hoteit turned on almost every appliance in the house (4 ACs, stove,. . .) to test the peak load of 5.44 kW (actual consumption is much less). This is what one must do when there is no or little grid, like in Puerto Rico, a failing US territory that seems to be comparable to Lebanon.

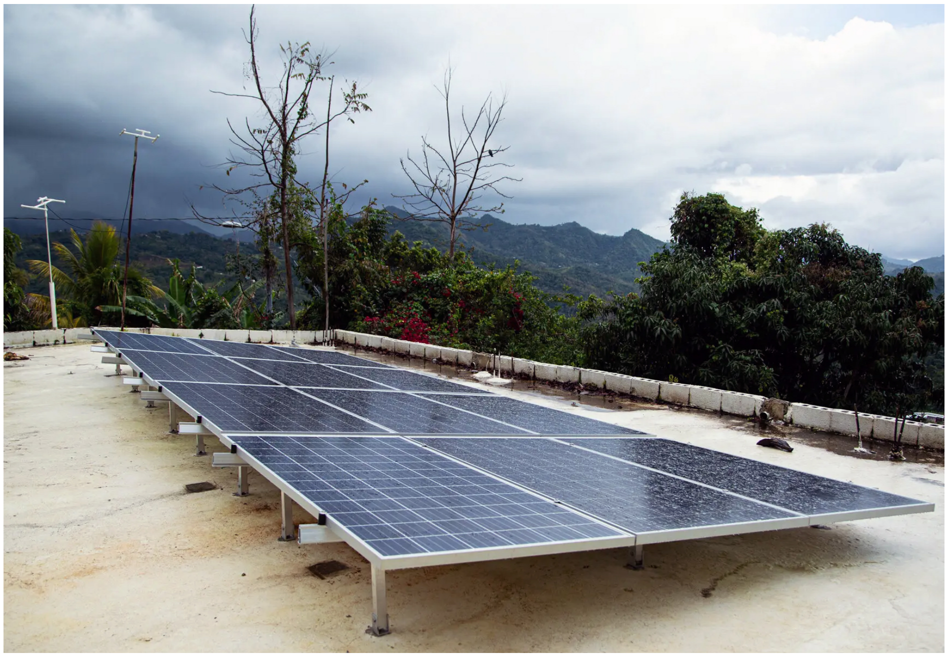

Fig. S40. Puerto Rico's electric grid fails regularly, sometimes islandwide with grave consequences to human life. An average solar PV system costs $27,000 there, and is out of reach for most people. Today, these systems provide only 2.5% of the island's electricity. To makes things worse, Luma Energy, the Canadian-American private utility company that took over the grid in Puerto Rico, wants to charge people for generating their own electricity. The solar panels that the nonprofit Casa Pueblo installed on the roof allow Mr. Molina's oxygen machine to keep running when the grid fails. Image source and narrative(35).

In summary, the capacity factors of the PV arrays in Austin, Càceres and Doueir (average for the Austin and Doueir locations, and 20% above the average in Càceres according to the solar atlas) have defined their physical performance *and* economics that follows from physics, not the other way around. These arrays were produced inside of the giant fossil fuel cocoon, using the conceivably most energy- and ultra clean water-intensive industrial processes that made them the 99.99999% pure pinnacle of all human industrial technologies. These arrays cannot possibly replace the fossil/nuclear/hydro electricity, carbon and heat that created them in the first place. Our strong fear is that when the current giant, mostly fossil fuel infrastructure deteriorates enough(1, 4, 36, 37), industrial capability of producing many GW_p per year of solar photovoltaics will cease to exist in 10-20 years or faster. We are afraid that the year 2022 is a preview of more things to come in this most difficult decade. Based on Patzek's 20 years of researching global power transitions and teaching a graduate course on this subject, it is obvious that renewables can never replace the current electrical power mix for humanity. They *can*, however, amend and modify this power mix in the developed countries and slash it down to 40-50% of its current capacity. We really need to start talking about serious curtailments of power use and changes in lifestyles of affluent societies. Happily, the authors of this paper, their friends and families, including children and grandchildren, already live this new reality.

Figure S41 compares the prices per W_p of bare PV modules, the turnkey costs of the Austin and Càceres systems, as well as estimates of the costs of US rooftop systems from the Solar Energy Industries Association (SEIA), and of the UK array costs from the Department for Business, Energy & Industrial Strategy. It is obvious that post 2006, the module prices and the total costs of PV systems have diverged. One key reason has been module dumping by China in order to bankrupt foreign competitors. While this Chinese trade policy has largely succeeded, the current trend of module pricing may be ending because of the changed geopolitical situation and quadrupling of the transportation costs.

In this paper, we have enumerated the shortfalls of the capacity factor. We do *not* suggest that all questions about an electricity generation plant can be answered with the capacity factor alone. One must also consider several aspects peculiar to the project location, size and role to make an informed decision. The complex modeling machinery used to obtain best projections and make what would be called an informed decision is rarely understood by diverse audiences and stakeholders, including policy makers. These complex models can backfire by implying that we have more certainty about their numerous inputs and predictions than can be reasonably assumed. Capacity factor is straightforward, delivers the message and can be easily understood and calculated. Capacity factor encapsulates the physics of electricity generation and provides a simple insight about average power that may be produced by an electricity source and about stability of the grid connected to this source. However improved, renewables have low capacity factors, suffer from lack of electricity storage, and are produced with large outlays of fossil energy and electricity.

One of the many dangers of using complex economics is obvious from reading this(38) most erudite, thorough paper with second order effects that simply cannot escape the multiple traps of classical macro- and micro-economics. Simplistically, if energy that powers the world accounts only for a few percent of an economy, and all economies go through a series of quasi-static equilibrium states, no current economic theory can show what we already see in real world: an impending crash of

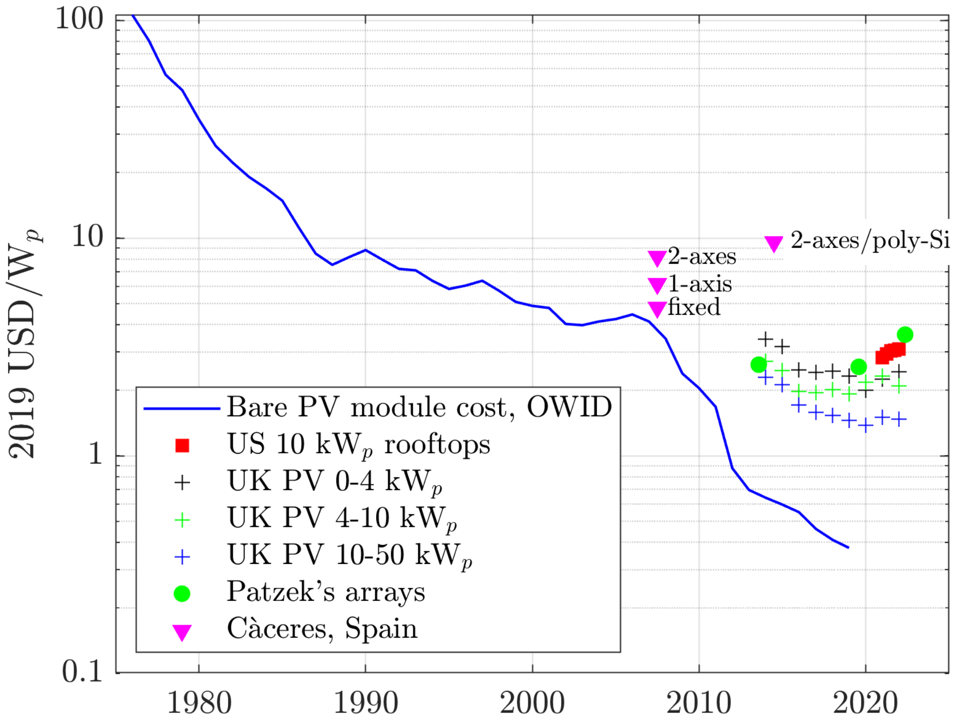

Fig. S41. Bare PV module costs according to Our World in Data. The post 2006 PV module dumping by China is obvious. In order to dominate the global markets, China has been very good at hiding the real module costs. However, other costs dominate residential installations in the US and Spain. The rooftop arrays are much cheaper to install than ground arrays, yet their 2022 average cost per W_p, after the federal rebate, has increased to over 10 times the module cost. The UK array costs are based on the mean prices of hundreds to thousands of installations per month, https://www.gov.uk/government/statistics/solar-pv-cost-data. In 2022, Patzek estimates that the installation cost of his 2019 array would have been above \$3.60/W_p after the federal rebate.

the global economy with the direst consequences(†).

This crash will be caused(40) by the deepening social inequality and increasing dynamic disequilibrium between the high biophysical power demand and ability to deliver this power millisecond-by-millisecond by the overtaxed electric and heat power systems around the world. From our point of view, the leading economic theories are akin to saying that if oxygen constitutes only 20 mol% of air, removing it from the air altogether will result in at most 50% deterioration of human performance after all second order, quasi static effects have been accounted for.

It is important to remind the Readers that most energy projects rely on a levelized cost of energy (LCOE), which implicitly relies on an estimate of capacity factor. In short, all economics of electricity generation depends on capacity factor. LCOE hides the fundamental difference between fossil and renewable power plants. In a fossil power plant, a 5-year investment is followed by 30-50 years of high rate output and earnings offset by high fuel and maintenance costs. In a renewable power plant, an instantaneous high investment is followed by 15-25 years of low rate output and earnings, with usually low maintenance costs, unless desalinated water is used to wash dust-covered arrays every two weeks in desert locations. Herein we emphasize the versatility and insights provided by a simple analysis of capacity factor, a number that a stakeholder or policymaker can easily calculate. Our main point is to clarify the overall performance of most sources of electrical power and avoid unrealistic overestimates that are frequently reported.

We are keenly aware that several economic aspects play a role in the decision to deploy a certain project. However, economic analysis has its own shortfalls. Whenever we compare the prices of renewable and fossil electricity systems, we tend to forget that these systems are fundamentally different. The real-life performance of all major electricity sources is based on their physical limitations that will not change because the market is favorable. Potential material limitations, such as that of copper, are mentioned in one sentence only, because they are outside of the scope of this paper. However, these potential material constraints will force choices that guarantee the optimal performance of any device to be installed. The capacity factor tells us how much we can rely on a source and the role that it can play.

A great example is the current war in Ukraine and the pressure on certain countries to cut ties with Russia. The UK answered these challenges by setting up a plan that will expand nuclear power capacity and give the *energy fields of the North*

(†) The economist Joseph Stiglitz, a Nobel laureate, said(39) the market failed to accurately price in the risk – however unlikely it may have seemed at the time – that Russia could decide to reduce or withhold gas to apply political pressure. It would be like figuring the costs of building a ship without including the cost of lifeboats. "They didn't take into account what could happen," Mr. Stiglitz said.

Sea a new lease of life. At the same time, the UK continues to invest heavily in renewables. In practice, the most expensive energy is the energy that we do not have. Nuclear electricity will be the backbone of a robust energy transition in the UK. Points like this are becoming clear to many European governments; we need to have robust systems in place to aim for an effective energy transition subject to military and economic wars. Economics coupled with low performance systems cannot secure a sound Energiewende, as Germany for example has discovered(31), see fig. S1. This summer in Germany, dirty, brown coal-fired power plants that were slated for retirement are being restarted to divert natural gas into storage supplies for the winter.

Two recent papers proposed a sophisticated analysis of energy returned on investment (EROI) for the static(41) and dynamic(42) grid penetration by the variable output renewables. Here is a summary of the latter paper: "A novel methodology is developed to dynamically assess the energy and material investments required over time to achieve the transition from fossil fuels to renewable energy sources in the electricity sector. The obtained results indicate that a fast transition achieving a 100% renewable electric system globally by 2060 consistent with the Green Growth narrative could decrease the EROI of the energy system from current ∼12:1 to ∼3:1 by the mid-century, stabilizing thereafter at ∼5:1. These EROI levels are well below the thresholds identified in the literature required to sustain industrial complex societies. Moreover, this transition could drive a substantial re-materialization of the economy, exacerbating risk of unavailability in the future of some minerals. Hence, the results obtained put into question the consistence and viability of the Green Growth narrative."

The results of(42) were used in(43) to perform a detailed, sophisticated economic analysis of green transitions. The results were summarized as follows: "A number of papers in the field of net energy analysis have argued that declines in energy return on investment (EROI) could lead to increasing energy prices and a fall in economic growth. This paper develops a model (TranSim) which can simulate the economic and financial implications of an energy technology transition involving a reduction in EROI, by combining the stock-flow consistent (SFC) approach with an input-output (IO) model. The TranSim model has the following key features. First, it includes three firm sectors, that produce energy, capital, and other (non-energy, non-capital) goods. Second, an IO model and an Almost Ideal Demand System are integrated into the SFC model. Third, capital vintages have embedded levels of labour productivity and intermediate good requirements that depend on the economic conditions in the period the vintage was produced. Simulations are characterised by an initial increase in output (due to higher investment), followed by periods of recession and below trend growth (due to price inflation and changes to the functional income distribution). The negative effects associated with the transition – recession, stagnation, stagflation, increasing inequality and asset stranding – are positively related to the capital intensity of green energy production and reductions in EROI."

One of the many economic outcomes of different green energy transition scenarios in this paper is shown in Figure S42. After the initial stimulation of an economy by the high initial capital spending on a green transition, this economy goes invariably into contraction caused by the low capacity factor of renewables (their low EROI). So, for the rich countries, the only long-term answer to climate change is to continue with a green energy transition *and* consume much less fast.

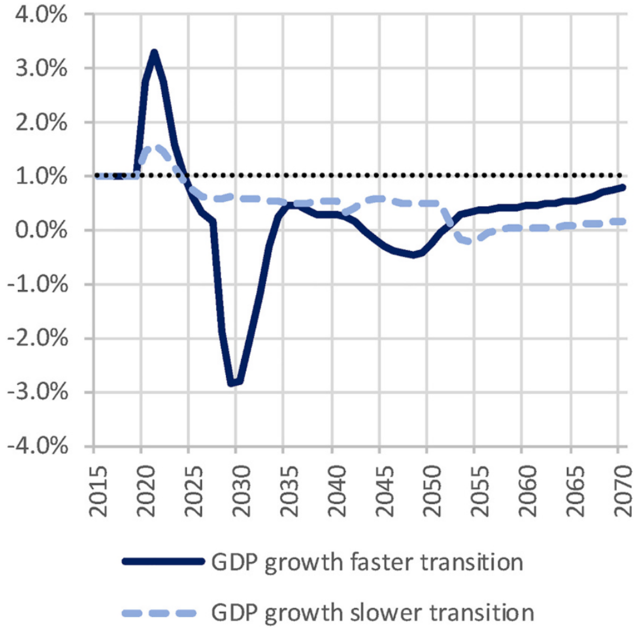

Fig. S42. Figure 29 in(43) compares growth rates of an economy during the faster and slower transitions to a green economy. The main difference between the slower/faster transitions is that in the slower transitions the variations in particular variables (*e.g.* energy prices, unemployment, output growth) are smaller, but last for a longer period of time. However, the smaller variations but longer durations approximately offset each other, so that by the time the economy has returned to a steady state it is in roughly the same place (*e.g.* with regards to the level of output, unemployment, prices, the wage profit split, etc.). Figure reproduced with a permission from the authors.

SI Dataset S1 (Algorithms)

Matlab algorithms. All algorithms developed by the authors and data collection from previously specified sources can be found at:

N.Bolson.*Data and codes for Capacity Factor: Values and Implications.* Available at https://doi.org/10.5281/zenodo.6565027. Deposited 21 May 2022.

References

1. TW Patzek, Thermodynamics of the corn-ethanol biofuel cycle. *Crit. Rev. Plant Sci.* **23**, 519–567 (2004).
2. TW Patzek, D Pimentel, Thermodynamics of energy production from biomass. *Crit. Rev. Plant Sci.* **24**, 329–364 (2005).
3. D Pimentel, TW Patzek, Ethanol production using corn, switchgrass, and wood; biodiesel production using soybean and sunflower. *Nat. Resour. Res.* **14**, 67–76 (2005).
4. TW Patzek, How can we outlive our way of life? in *20th Round Table on Sustainable Development of Biofuels: Is the Cure Worse than the Disease?* (OECD, Paris), pp. 1– 49 (2007) http://www.oecd.org/sd-roundtable/papersandpublications/40225820.pdf.
5. TW Patzek, Thermodynamics of Agricultural Sustainability: The Case of US Maize Agriculture. *Crit. Rev. Plant Sci.* **27**, 272 – 293 (2008).
6. AW Baum, TW Patzek, M Bender, S Renich, W Jackson, The Visible, Sustainable Farm: A Comprehensive Energy Analysis of a Midwestern Farm. *Crit. Rev. Plant Sci.* **28**, 218–239 (2009).
7. M Giampietro, SG Bukkens, Knowledge claims in European Union energy policies: Unknown knowns and uncomfortable awareness. *Energy Res. & Soc. Sci.* **91**, 102739 (2022).
8. TF Allen, Starr. TB 1982. Hierarchy: perspectives for ecological complexity (1983).
9. R Rosen, *Life itself: a comprehensive inquiry into the nature, origin, and fabrication of life.* (Columbia University Press), (1991).
10. P Bak, Self-organized criticality: A holistic view of nature in *Complexity, Metaphors, Models and Reality*, eds. G Cowan, D Pines, D Meltzer. (Addison-Wesley Publishing Co., Santa Fe Institute), Vol. XIX, pp. 477–496 (1994).
11. G Box, Robustness in the Strategy of Scientific Model Building in *Robustness in Statistics*, eds. RL LAUNER, GN WILKINSON. (Academic Press), pp. 201–236 (1979).
12. A Zellmer, T Allen, K Kesseboehmer, The nature of ecological complexity: A protocol for building the narrative. *Ecol. Complex.* **3**, 171–182 (2006).
13. EIA, International (2020).
14. World Bank, Electricity production from oil sources (2020).
15. World Bank, Electricity production from coal sources (2020).
16. World Bank, Electricity production from natural gas sources (2020).
17. EIA, World installed coal-fired generating capacity (2019).
18. EIA, World installed natural-gas-fired generating capacity (2019).
19. EIA, World installed liquids-fired generating capacity (2019).
20. BK Sovacool, P Schmid, A Stirling, G Walter, G MacKerron, Differences in carbon emissions reduction between countries pursuing renewable electricity versus nuclear power. *Nat. Energy*, 1–8 (2020).
21. IMF, World economic outlook - real GDP growth (2020).
22. IMF, World economic outlook, (IMF), Technical report (2012).
23. CA Greene, et al., The climate data toolbox for Matlab. *Geochem. Geophys. Geosystems* **20**, 3774–3781 (2019).
24. WNA, Nuclear power in Japan (2019).
25. Japan Times, The stalled restart of idled reactors (2019).
26. S Johnson, Middle East countries plan to add nuclear to their generation mix (2018).
27. WNA, Emerging nuclear energy countries (2020).
28. EUROPEAN COMISSSION-JRC, Photovoltaic geographical information system (pvgis) (year?).
29. SAS Eddington, *The Nature of the Physical World.* (Cambridge University Press, New York), (1953).
30. CW King, JD Rhodes, J Zarnikau, The Timeline and Events of the February 2021 Texas Electric Grid Blackouts, (The University of Texas at Austin, Energy Institute), Report by a committee of faculty and staff (2021).
31. Editorial, Europe must not backslide on climate action despite war in Ukraine. *Nature* **607** (2022).
32. A Louwen, WGJHM van Sark, APC Faaij, REI Schropp, Re-assessment of net energy production and greenhouse gas emissions avoidance after 40 years of photovoltaics development. *Nat. Commun.* **7**, 1–9 (2016).
33. V Fthenakis, et al., PHOTOVOLTAIC POWER SYSTEMS PROGRAMME, Life Cycle Inventories and Life Cycle Assessments of Photovoltaic Systems, (INTERNATIONAL ENERGY AGENCY, Paris), IEA PVPS Task 12, Subtask 20, LCA IEA-PVPS T12-02:2011 (2011).
34. N Bolson, T Patzek, Evaluation of Rwanda's Energy Resources. *Sustainability* **14**, 6440 (2022).
35. Coral Murphy Marcos, Solar Power Offers Puerto Ricans a Lifeline but Remains an Elusive Goal (www.nytimes.com/2022/05/09/business/energy-environment/puerto-rico-solar-power.html) (2022).
36. N Bolson, M Yutkin, W Rees, T Patzek, Resilience rankings and trajectories of world's countries. *Ecol. Econ.* **195**, 107383 (2022).

37. N King, A Jones, An Analysis of the Potential for the Formation of 'Nodes of Persisting Complexity'. *Sustainability* **13** (2021).
38. DR Baqaee, E Farhi, The Macroeconomic Impact of Microeconomic Shocks: Beyond Hulten's Theorem. *Econometrica* **87**, 1155–1203 (2019).
39. Patricia Cohen and Melissa Eddy, Germany Hopes to Outrace a Russian Gas Cutoff and Bone Cold Winter (www.nytimes.com/2022/07/15/business/economy/germany-russia-natural-gas.html) (2022).
40. S Motesharrei, J Rivas, E Kalnay, Human and nature dynamics (HANDY): Modeling inequality and use of resources in the collapse or sustainability of societies. *Ecol. Econ.* **101**, 90–102 (2014).
41. C de Castro, I Capellán-Pérez, Standard, Point of Use, and Extended Energy Return on Energy Invested (EROI) from Comprehensive Material Requirements of Present Global Wind, Solar, and Hydro Power Technologies. *Energies* **13** (2020).
42. I Capellán-Pérez, C de Castro, LJ Miguel González, Dynamic Energy Return on Energy Investment (EROI) and material requirements in scenarios of global transition to renewable energies. *Energy Strateg. Rev.* **26**, 100399 (2019).
43. A Jackson, T Jackson, Modelling energy transition risk: The impact of declining energy return on investment (EROI). *Ecol. Econ.* **185**, 107023 (2021).
